# Connexin 36 Mediated Intercellular Endoplasmic Reticulum Stress Transmission Induces SSTA Resistance in Growth Hormone Pituitary Adenoma

**DOI:** 10.7150/ijbs.86736

**Published:** 2024-01-01

**Authors:** Zihan Wang, Zhuowei Lei, Quanji Wang, Qian Jiang, Zhuo Zhang, Xiaojin Liu, Biao Xing, Sihan Li, Xiang Guo, Yanchao Liu, Xingbo Li, Yiwei Qi, Kai Shu, Huaqiu Zhang, Yimin Huang, Ting Lei

**Affiliations:** 1 Department of Neurosurgery, Tongji hospital of Tongji medical college of Huazhong University of Science and Technology, Wuhan, 430030, China.; 2 Sino-German Neuro-Oncology Molecular Laboratory, Tongji hospital of Tongji medical college of Huazhong University of Science and Technology, Wuhan, 430030, China.; 3 Hubei Key Laboratory of Neural Injury and Functional Reconstruction, Huazhong University of Science and Technology, Wuhan, 430030, China.; 4 Department of Orthopedics, Tongji hospital of Tongji medical college of Huazhong University of Science and Technology, Wuhan, 430030, China.

**Keywords:** Growth hormone-secreting pituitary adenoma, Somatostatin, Endoplasmic reticulum stress, Connexin 36, Gap junctions, Quinine

## Abstract

Somatostatin analogues (SSTA) are first-line pharmacological treatment choice for acromegaly, which received satisfying tumor shrinkage and normalization of growth hormone. However, there are still patients unresponsive to SSTA, and the underline mechanism remains unknown. Besides, there is no evidence regarding the role of endoplasmic reticulum stress (ERS) and its transmission in SSTA resistance, which also require investigation. Primary growth hormone adenoma cells and cell lines were treated with SSTA; autophagy double-labeled LC3 (mRFP-GFP) adenovirus transfection, flow cytometry sorting, western blotting, calcium imaging as well as immunofluorescence staining were used to determine ERS and autophagy signal transmission; xenograft and syngeneic tumor *in vivo* model were exploited to confirm the ERS signal transmission mediated effect. Our results revealed that SSTA induces ERS in pituitary growth hormone (GH) adenoma cells. The ERS signals can be intercellularly transmitted, leading to less responsible to SSTA treatment. Moreover, SSTA stimulates inositol triphosphate (IP_3_) elevation, mediating ERS intercellular transfer. In addition, connexin 36 tunnels ERS transmission, and its blocker, Quinine, exhibits a synergistic effect with SSTA treating GH adenoma. Our study provided insight into ERS intercellular transmission mediated SSTA resistance, which could be translated into clinical usage to improve SSTA efficiency in GH adenoma treatment.

## Introduction

GH adenoma is a relatively rare disease that causes acromegaly, the excessive growth hormone and its target hormone insulin-like growth factor 1 (IGF-1) may result in a wide array of comorbidities 1, 2. Besides, GH adenoma can be detrimental by invasiveness or mass effect intracranially 3. Current strategies against GH adenoma mainly include pituitary surgery, medical therapy and radiation therapy 4. Pituitary surgery is the first-line options for most patients 5. Medical therapy and radiation therapy generally represent second-line and third-line options, and especially for patients who are not in remission postoperatively 6. Somatostatin receptor ligands (SRLs) or somatostatin analogs (SSTA) are first-line pharmacological treatment choices for acromegaly, which received satisfying tumor shrinkage and normalization of growth hormone. While however, first-generation SSTA only make 30%-40% unselected patients achieve biochemical control 7, and only 20% patients who are not controlled on first-generation SSTA can achieve biochemical control on second-generation SSTA 8, 9. Although several markers have been revealed as potential indicators of SSTA resistance 10, 11, the underline mechanism remains unknown.

In general, previous studies illustrated that SSTA binds to somatostatin receptor (SSTR) on cells and further activates SSTR-associated Gαi inhibitory proteins leading to suppression of downstream GPCR signaling, which plays a crucial role in hormone release and cell proliferation 12. Our group has investigated the pivotal role of GPCR signaling in pituitary adenoma since the 1990s. In addition, this suppression effect mainly causes decreased intracellular cAMP and Ca^2+^ signals and up-regulation of IP_3_ 13, 14. While little is known regarding the specific mechanism of SSTA on pituitary adenoma cell suppression.

ERS is a cellular response to misfolded and unfolded protein aggregation and dysregulation of calcium homeostasis in the lumen of the endoplasmic reticulum (ER) that activates signaling pathways 15. ERS can induce the expression of glucose-regulated protein GRP78, GRP94, and other endoplasmic reticulum molecular chaperones to produce protective effects but also independently induce endogenous apoptosis, which ultimately affects the fate of stressed cells, such as apoptosis or adaptation 16, 17. A previous critical study uncovered that ERS could be transmitted intercellularly 18. There is no study elaborating the effect of SSTA on ERS or its potential role in therapy resistance.

## Materials and Methods

### Human pituitary adenoma samples

Between June 2021 and November 2022, 11 growth hormone pituitary adenoma samples and 5 nonfunctional pituitary adenoma samples were collected from patients who underwent surgery at the Department of Neurosurgery, Tongji Hospital of Tongji Medical College, Huazhong University of Science and Technology. Samples were dissected into two parts for primary cell culture and tissue fixation, respectively. Generally, we included 6 females and 10 males, with a mean age of 45.8 years (range 21-67 years) at the time of surgery. This work was approved by the ethical committees at Tongji Hospital, Tongji Medical College, Huazhong University of Science and Technology. Written informed consent was obtained from all patients.

According to previous studies 19, 20, resistance to SSTA therapy could be defined as: (1) nadir OGTT-GH greater than 1.0μg/L and IGF-I levels above the normal range adjusted for age and gender; and (2) tumor shrinkage less than 20% compared to baseline volume after at least 12 months of treatment with adequate dosages.

### Cell lines and primary cell culture

GH3 (ATCC, CCL-82.1^TM^), GH4 (ATCC, CCL-82.2^TM^) cell line was purchased from the American Type Culture Collection without mycoplasma contamination (Mycoplasma Stain Assay Kit, Beyotime, cat no. C0296, China). GH3 and GH4 cells were routinely maintained in DMEM medium (Gibco, Thermo Fisher, Waltham, MA, USA) containing 10% fetal bovine serum (Gibco, Thermo Fisher, Waltham, MA, USA), 1% streptomycin, and 1% penicillin and maintained at 37°C in a humidified atmosphere of 95% air and 5% CO2. The medium was changed every 3 days.

On the first day, the fresh pituitary tumor tissue sample collected during surgery was put into a 50mL centrifuge tube soaked in physiological saline. Then, the tube was put in ice in a heat preservation bucket. Heat preservation bag was quickly transported to the laboratory and placed in a biological safety cabinet. A surgical blade was used to cut the tumor specimens into small tissue sections measuring <1 mm^3^ in a 100-mm diameter petri dish. Then, they were rinsed with PBS and centrifuged at 1,200 r/min for 4 min in a 15mL centrifuge tube, and the supernatant was discarded. Then, 4mL 0.25% trypsin was added into the centrifuge tube. The centrifuge tube was put in the incubator shaker, with a temperature of 37°C and a speed of 80 rpm, and the tube was shaken for 30 minutes. Then the tube was centrifuged at 1,200 r/min for 4 min, and the supernatant was discarded. 2 mL DMEM medium with 10% fetal bovine serum, 1% streptomycin, and 1% penicillin was added into the tube after mixing evenly, and the tissue fluid was filtered with a 40μm filter to get the cell suspension. Then, the cell suspension was transferred into a 60mm diameter petri dish. The primary cells were cultured in an incubator at a constant temperature of 37°C in a humidified atmosphere of 95% air and 5% CO2 for 24 h, and the cells were rinsed with PBS, and a fresh medium was added. Identification of growth hormone adenoma primary cells was shown in Figure S1.

### Tumor xenograft experiments

For in vivo assays, GH3 and GH4 cell xenograft models were performed. Briefly, nude mice were randomly divided into several groups and given 1 × 10^7 cells by subcutaneous injection in their right axilla. Tumor sizes were measured and recorded every three days after injection. Tumors were extracted 14 days after the injection, and the tumor weights were measured. Tumor samples were formalin-fixed and paraffin-embedded. The treatment started from the next day of tumor inoculation, which daily intraperitoneal injection of OCT (30μg/kg), PST (30μg/kg), 4-PBA (250mg/kg), CBX (100mg/kg), or Quinine (50mg/kg) in 200μL PBS was performed for each group, respectively. An equal volume of physiological saline was injected daily as a control.

### Drug treatment

For somatostatin analogs treatment in vitro, octreotide (200 nM; MCE, Shanghai, China) or pasireotide (200nM; MCE, Shanghai, China) dissolved in PBS was added to the culture medium. For endoplasmic reticulum stress inducement treatment in vitro, Tunicamycin (1μg/mL; Aladdin, Shanghai, China) dissolved in DMSO was added to the culture medium. For endoplasmic reticulum stress inhibitor treatment in vitro, 4-Phenylbutyric acid (500μM; MCE, Shanghai, China) dissolved in PBS was added to the culture medium. For nonspecific gap junction blocker treatment in vitro, Carbenoxolone disodium (100μM; MCE, Shanghai, China) dissolved in PBS was added to the culture medium. For IP3-R blocker treatment in vitro, 2-Aminoethyl diphenylborinate (100μM; MCE, Shanghai, China) dissolved in DMSO was added to the culture medium.0.02% DMSO was used as a control. For specific CX36 blocker treatment in vitro, Quinine (100μM; MCE, Shanghai, China) dissolved in DMSO was added to the culture medium. 0.05% DMSO was used as a control. Cells were harvested 24h after drug treatment for further examinations.

Daily intraperitoneal injection of OCT (30μg/kg), PST (30μg/kg), 4-PBA (250mg/kg), CBX (100mg/kg), or Quinine (50mg/kg) in 200μL PBS was performed for nude mice or wistar rats in the separate treatment group. An equal volume of physiological saline was injected daily as a control.

### ELISA

Growth hormone in the supernatant representing the secreting level of Iceland ls, IP3 in cell lysate representing IP3 level in cells was determined as follows. Growth hormone and IP3 concentrations were measured via ELISA Kits for GH (Elabscience, Wuhan, China) and IP3 (Elabscience, Wuhan, China). ELISA experiments were performed according to the manufacturer's instructions. The supernatant was centrifuged before use and stored at -80°C. Ultrasonic pyrolysis was used for lytic cell suspension (1x10^6 cells/100μL PBS) to get the cell lysate, and the lysate was stored at -80°C. IP3 concentrations (pg/mL) were normalized to cell counts in each group (1x10^6 cells/100μL PBS).

GH concentrations (ng/mL) were normalized to cell counts at the beginning of each group (1x10^6 cells/2mL medium).

### Flow cytometry

The cell cycle progression and apoptosis were investigated using flow cytometry. The cell cycle was investigated using a PI Cell Cycle detection kit (Yeasen, Shanghai, China). The cells were digested with 0.25% trypsin, then centrifuged at a speed of 1000rpm/min for 5mins, and the supernatant was discarded. The cells were washed one time with PBS precooled. The cells were gently mixed with 70% ethanol precooled and fixed overnight at 4°C. The cells were centrifuged at a speed of 1000rpm/min for 5mins and resuspended with 1mL precooled PBS. And then, it was centrifuged at a rate of 1000rpm/min for 5mins. 10μL propidium iodide storage solution and 10μL RNase A solution were added to the 0.5mL staining buffer. Each cell sample was added with 0.5mL propidium iodide staining solution configured, and the suspended cells were gently mixed. Then the cells were incubated in the dark at 37°C for 30mins. The cells were filtered with a 400-mesh sieve and detected by flow cytometryCytoFLEX V5-B5-R3 Flow Cytometer (13 Detectors, 3 Lasers) (Beckman, California, USA).

The cell apoptosis was investigated using an Annexin V-FITC/PI apoptosis detection kit (Yeasen, Shanghai, China). The cells were digested with 0.25% trypsin without EDTA and centrifuged at 4°C for 5 min. The cells were washed with precooled PBS and each time was centrifuged at 4°C for 5 min. 1-5x10^5 cells were collected. PBS was abandoned, and 100μL 1 × Binding Buffer was added to resuspend the cells. 5μL Annexin V-FITC and 10μL PI Staining Solution were added and mixed gently. Then the cells were incubated in the dark at room temperature for 10-15 mins. 400μL 1 × Binding Buffer was added, mixed, and placed on the ice. The cells were filtered with a 400-mesh sieve and detected by flow cytometry. The data were analyzed using Flowjo v10 software.

### Colony formation assay

Cells were seeded into 6-well plates (1000 cells/well). After 24h, the medium was changed to a fresh complete medium, including the drug of each treatment group. Cells were maintained for two weeks; the medium was renewed once per week. The colonies were visualized by crystal violet staining after fixation with 4% paraformaldehyde.

### Autophagosome labeling

The autophagosomes of GH3 cells were labeled by mRFP-GFP-LC3, which was transfected into GH3 cells by adeno-associated virus. The mRFP-GFP-LC3 adenoviral particles were purchased from HanBio (Shanghai, China). Cells were incubated with adenoviral particles for 24h, and then the adenoviral particles were removed, the cells were cultured for another 24 h. Autophagosomes were observed using laser confocal microscope (OLYMPUS FV1000).

### Plasmid construction and transfection

Lentiviruses of shRNAs were constructed by Genechem (Shanghai, China). The target sequences are as follows:

GJD2:

sh1: GCAGCACTCCACTATGATTGG; sh2: GCATTTGTGTGGTGCTCAATC;

sh3: GGAGCAAGCGAGAAGATAAGA.

SSTR2:

sh1: CCAGCCCTTAAAGGCATGTTT; sh2: CCCTATCCTATATGCCTTCTT;

sh3: GCAGTCCTCACATTCATCTAT.

SSTR5:

sh1: CGTCACCAACATCTACATTCT; sh2: CTTCACCGTCAACATCGTCAA;

sh3: CAACCAGTTCACCAGTGTCTT

Viral capsid plasmids (PSPAX 1μg and PMD2G 1μg) and plasmids (NC-shRNA 2μg or GJD2-shRNA 2μg) were added to 200μL opti-mem medium (Gibco, Thermo Fisher, Waltham, MA, USA); transfection reagents (Lipo3000 4μg and P3000 4μg) (Invitrogen, Thermo Fisher, Waltham, MA, USA) were added to 200μL Opti-MEM medium. The two solutions were mixed, and after standing for 20 minutes, the mixed solution was added to 293T cells; after 6-8 hours, the solution was changed with 2mL DMEM medium (10%FBS). The virus solution was collected 72 hours later, filtered with a 0.22μm filter, and added to GH3 or GH4 cells. The virus solution was changed with 2mL DMEM medium (10% FBS) after 6-8 hours. The stable transformation cell line was obtained by screening with medium containing 2.5mg/mL Puromycin 72 hours later for 3 days.

### 3D sphere formation experiment

To better simulate the shape of the tumor, 3D sphere formation experiment was conducted. Collagen, Type I, from rat tail (40125ES10 Yeasen, Shanghai, China) was used to configure gel on ice, referring to the instructions of rat tail collagen Ⅰ (supplementary material). The gel was 1:7.5 mixed with DMEM medium (10%FBS), and the mixed medium was added into a round bottom-low attachment 96-well plate (Corning, Kennebunk, USA) 200μL per well, then 5000 cells were added into each well, then the 96-well plate was centrifuged at 4°C at 1000g for 10 minutes to make the cell sphere. After 1 week incubation, the cell spheres were collected and stained using immunofluorescent method. And then, the spheres were observed using fluorescence microscope (Olympus, Tokyo, Japan).

### Contact co-culture experiment

To study the signal transmission of endoplasmic reticulum stress between cells, a contact co-culture experiment was designed. The cells stably expressing GFP fluorescent protein were used as donor cells, and the normal cells that did not express GFP fluorescent protein were used as recipient cells. The donor cells were stimulated with endoplasmic reticulum stress inducer Tm (1μg/mL) or SSTA drug OCT (200nM) for 24 hours, then the cells were digested with 0.25% trypsin and centrifuged at 1200rpm for 4 minutes, after resuspended, the donor cells were added into the recipient cells for co-culture for 24 hours. The expression of endoplasmic reticulum stress proteins in donor and recipient cells was observed by flow cytometry or fluorescence microscope after immunofluorescence staining.

### Cytosolic Ca2+ Assessment

Cytosolic Ca2+ levels were determined using Fura-4 AM (Yeasen, Shanghai, China) and Rhod-2 AM (40776ES50, Yeasen, Shanghai, China). Cells were loaded with Fura-4 AM or Rhod-2 AM in HBSS for 30 mins at temperature of 37°C and then the solutions were removed. Then, cells were washed with HBSS for 3 times to fully remove Fura-4 AM or Rhod-2 AM, and the cells were covered with HBSS for 30 mins at temperature of 37°C. Fluorescence images were obtained using an OLYMPUS CKX5 fluorescence microscope (Olympus, Tokyo, Japan).

### Statistical analyses

All data are expressed as the mean ± standard deviation. The program GraphPad Prism 9.0 was used for statistical analysis and chart drawing. Adobe Photoshop CC2018 was used for picture clipping. One-way ANOVA followed by Tukey's test was used for multiple comparisons. A non-paired t-test was used when two groups were compared. Statistical significance was considered to be indicated by a value of p < 0.05.

Other methods are described in the Supplementary Material.

## Results

### SSTA enhances pituitary adenoma cells ERS which mediates cell autophagy

**Figure 1:**
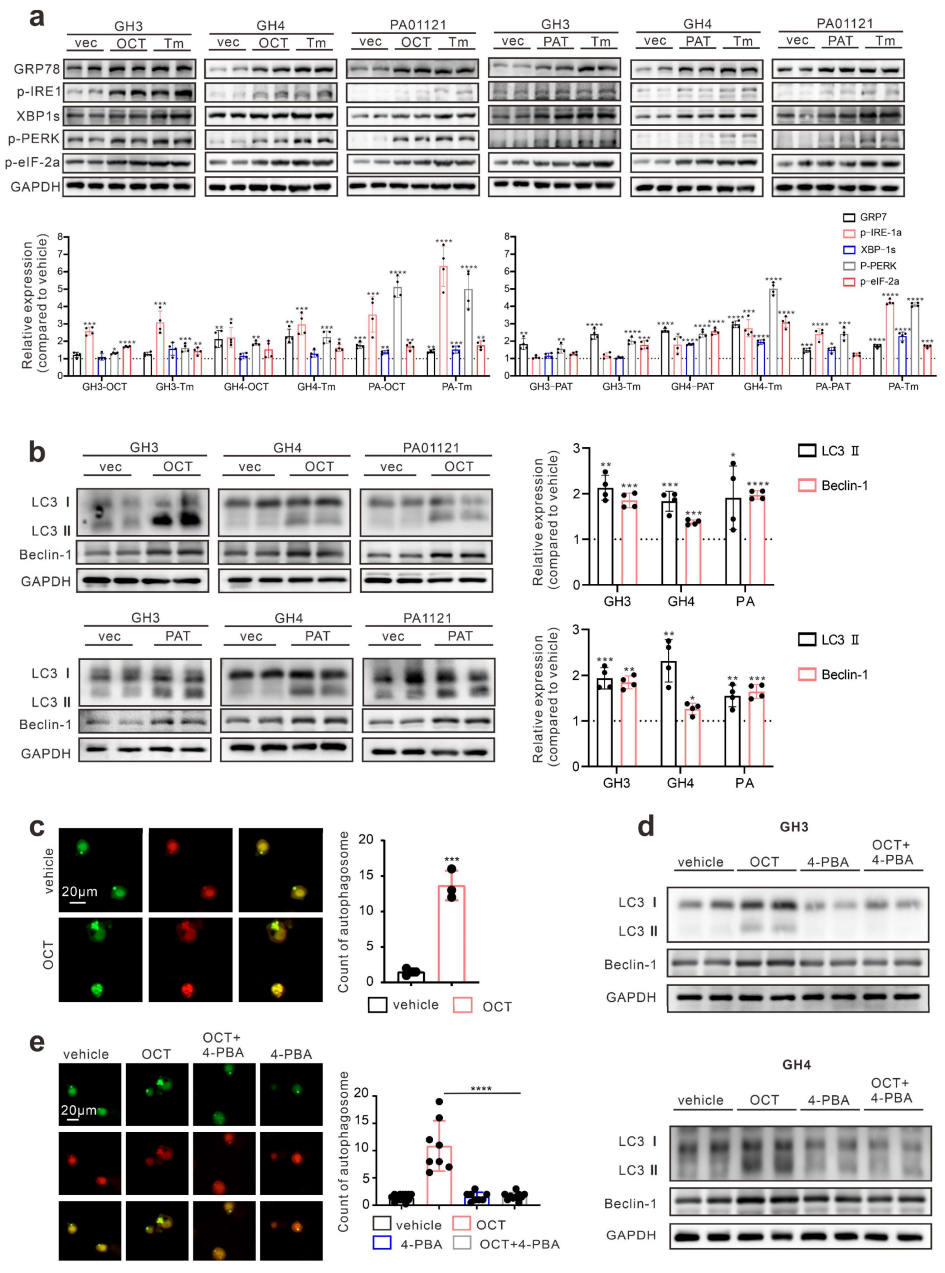
** SSTA enhances ERS of GH adenoma cells which mediates cell autophagy.** (a) Western blot of ERS proteins after treatment with either OCT (200nM), Tm(1.0μg/ml), or with PBS (vec) for 24h. Below bar graphs depicted the fold change of corresponding targets (normalized to GAPDH) of each group to vehicle. (b) Western blot of autophagy proteins after treatment with either OCT (200nM) or PBS (vec) for 24h. Below bar graphs depicted the fold change of corresponding targets (normalized to GAPDH) of each group to vehicle. (c) GH3 cells with mRFP-GFP labeled LC3 were treated with OCT (200nM) or PBS (vehicle) for 24 h, and the number of autophagosomes was counted under fluorescence microscope (***P=0.0006). (d) Western blot of autophagy proteins after treatment with either OCT (200nM), 4-PBA (500μM), OCT+4-PBA, or PBS (vehicle) for 24h. (e) GH3 cells with mRFP-GFP labeled LC3 were treated with either OCT (200nM), 4-PBA (500μM), OCT+4-PBA, or PBS (vehicle) for 24 hours, and the number of autophagosomes was counted under a fluorescence microscope (****P<0.0001). Data are shown as the mean ± SD of at least three independent experiments. Statistical analyses were conducted using one-way ANOVA and Student's *t* test.

First, we determined whether SSTA can induce ERS in pituitary adenoma cells. Pituitary adenoma cell lines GH3, GH4 and primary tumor cells were treated with octreotide (OCT) or pasireotide (PAT), ERS associated proteins were detected. We used Tunicamycin (Tm) as a positive control for inducing endoplasmic reticulum stress 21, 22. As shown in Fig. 1a, SSTA notably up-regulated the ERS level in pituitary adenoma cells. Since autophagy has been revealed as crucial downstream signaling of ERS 23, we further examined the autophagy of pituitary adenoma cells. As expected, SSTA drastically induced autophagy in pituitary adenoma cells (Fig. 1b). To further validate this, fluorescence-labeled LC3 virus was used to evaluate autophagy. SSTA indeed induced a higher density of autophagosomes (Fig. 1c). To investigate whether SSTA-induced ERS mediates autophagy, ERS was blocked by 4-Phenylbutyric acid (4-PBA), and we observed that autophagy induced by SSTA was remarkably attenuated by ERS inhibition (Fig. 1d-1e).

### ERS signals de-sensitize GH adenoma cells to SSTA and is heterogeneous inside the tumors

**Figure 2:**
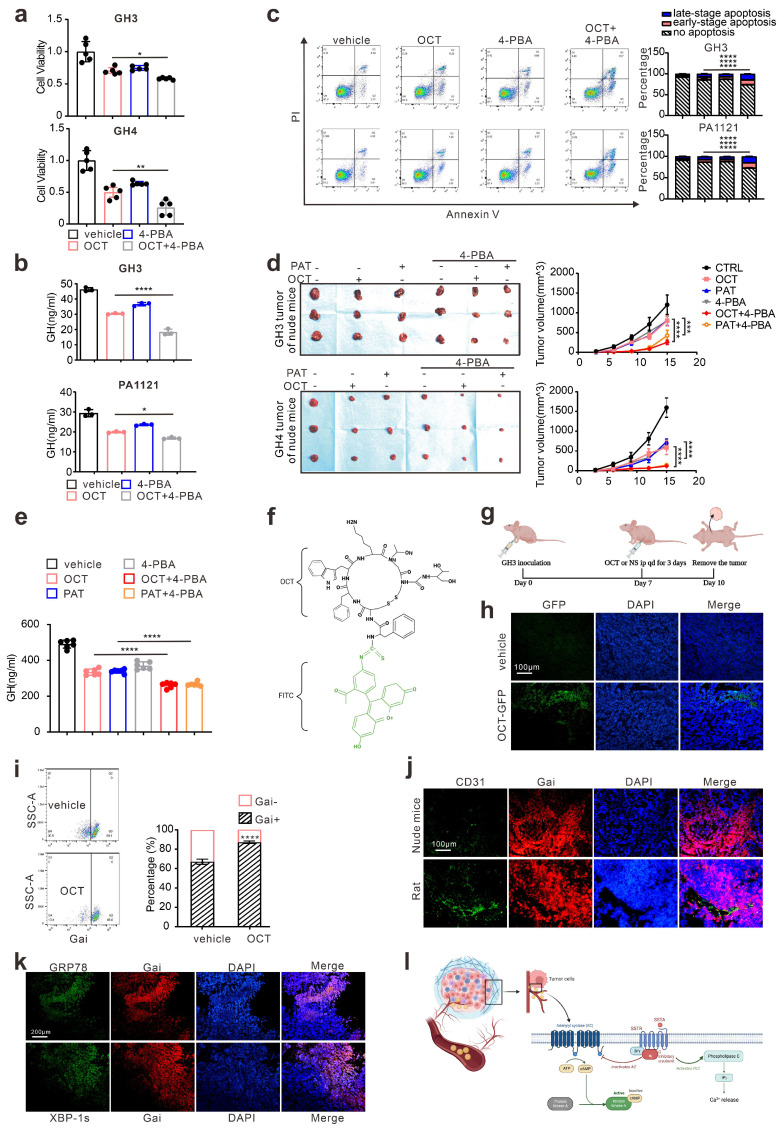
** ERS signal de-sensitize GH adenoma cells to SSTA and is heterogeneous inside the tumors.** (a) CCK-8 assay of the effect of OCT (200nM),4-PBA (500μM) and OCT+4-PBA (24h) (N=5, *P=0.0320; **P=0.0013). (b) ELISA assay of the effect of OCT (200nM), 4-PBA (500μM), OCT+4-PBA (24h) on GH concentration of supernatant (N=3, ****P<0.0001; *P=0.0199). (c) Annexin-V apoptosis flow cytometry of GH3 and human GH adenoma primary cells treated with either OCT (200nM), 4-PBA (500μM), OCT+4-PBA or PBS (vehicle) for 24h (N=3, ****P<0.0001). (d) GH3 and GH4 cells were used to form tumors under the axils of nude mice, the nude mice were injected intraperitoneally with either OCT (30μg/kg), PAT (30μg/kg), 4-PBA (250mg/kg), OCT+4-PBA, PAT+4-PBA or PBS (vehicle) for once a day. The tumor sizes were measured every 3 days (volume=0.5*length*width^2) (N=3, ****P<0.0001; ***P=0.0005). (e) ELISA assay of GH concentration in blood samples collected from the orbit of nude mice (N=5, ****P<0.0001). (f) The structure of fluoresce isothiocyanate labeled octreotide (FITC-OCT). (g) The process of tumor implantation, drug injection, and tumor removal in nude mice. (h) FITC-OCT distribution in xenograft of nude mice. (i) Flow cytometry of xenograft cells, the proportion of Gαi positive cells is calculated (N=3, ****P<0.0001). (j)(k) Immunofluorescence staining of sections of the xenografts. (l) Graphic abstract of drug action *in vivo*. Common theory believes that SSTA activates Gαi and act as suppressive signals to GPCR, in downstream, PLC and IP3 are upregulated in response to SSTA. Data are shown as the mean ± SD of at least three independent experiments. Statistical analyses were conducted using one-way ANOVA and Student's *t* test.

Since either ERS or its downstream autophagy has been revealed as crucial drug resistance pathways 24, we then verified the protective role of ERS in SSTA treatment. By using 4-PBA to block ERS 25, 26, we revealed the enhancement of SSTA-mediated suppression of cell viability, cell proliferation, and GH releases after ERS inhibition (Fig. 2a-2b, Fig. S2a-S2b). Moreover, ERS blocking resulted in a higher level of apoptosis in the presence of SSTA (Fig. 2c) and a more apparent blocking effect of the cell cycle in G0/G1 phase (Fig. S2c). To verify our observation *in vivo*, GH3 or GH4 pituitary adenoma cells were subcutaneously inoculated in the nude mice and SSTA with or without 4-PBA were injected intraperitoneally. Similar to *in vitro* observation, ERS inhibition and SSTA led to significant tumor shrinkage and slower tumor growth (Fig. 2d, Fig. S2d-S2e), as well as a greater decrease of GH release (Fig. 2e).

Drug distribution has long been discussed as an important cause for chemoresistance 27, 28. To determine the uneven distribution of SSTA in GH adenoma, we utilized the fluorescence-labeled octreotide (FITC-OCT) (Fig. 2f) to administrate GH3 cells inoculated nude mice (Fig. 2g). As expected, OCT unevenly distributed within the tumor (Fig. 2h). Moreover, since SSTA activates Gαi and act as suppressive signals to GPCR 13, 14, we then examined Gαi signals within tumor mass by flow cytometry. Indeed, OCT induced an increased level of Gαi positive cells, while still there are approximately 15% of the cells are Gαi negative (Fig. 2i). Furthermore, vessels were labeled by CD31, and we observed the aggregation of Gαi positive cells along the vascular structure (Fig. 2j). Furthermore, we examined the ERS-associated proteins in SSTA treated tumors. We found that ERS signals are also unequally spread. Its distribution area is similar to that of Gαi (Fig. 2k). Next, we assessed the uneven distribution of Gαi signals using 3D pituitary adenoma cell culture. Similar to *in vivo* experiments, the tumor spheres exhibit an uneven distribution of Gαi signals either in cell lines or in primary GH adenoma cells (Fig. S3). Taken together, our data illustrated that ERS has a protective effect on pituitary adenoma after SSTA treatment, and SSTA-mediated ERS signals are unevenly distributed among the tumor mass. The process of somatostatin analogs acting in GH adenoma is shown in Fig. 2l.

### ERS signals intercellular transmission induces pituitary adenoma resistance to SSTA

**Figure 3:**
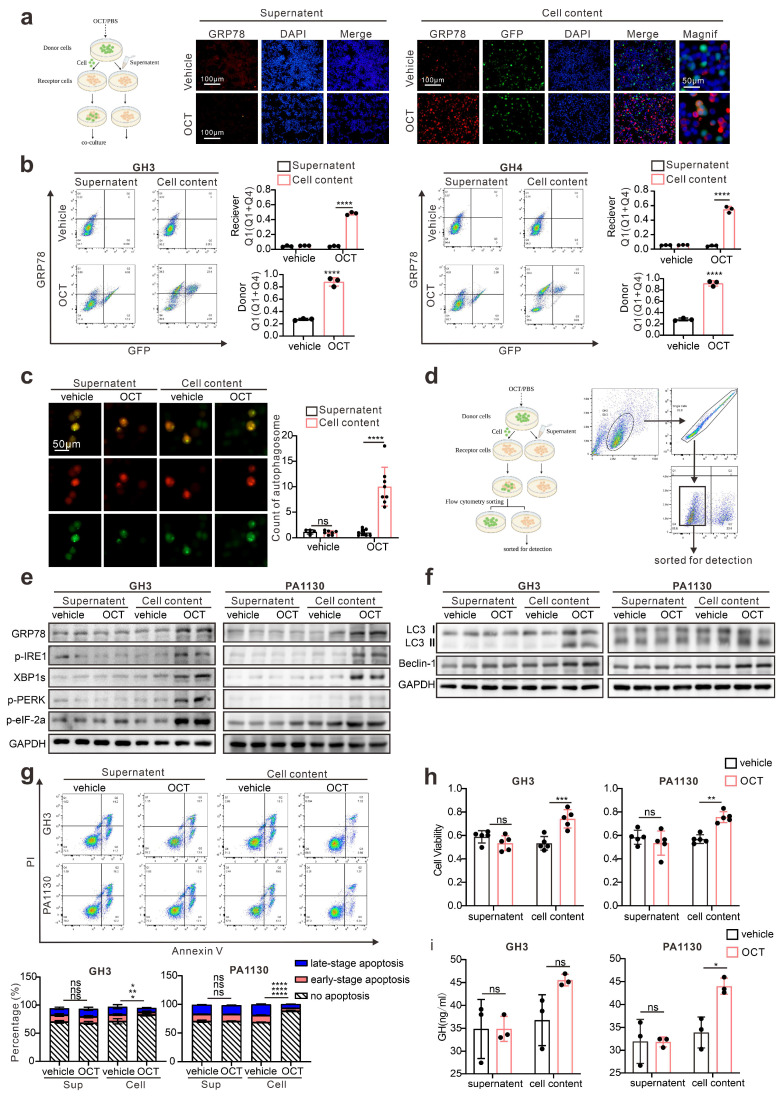
** ERS signals intercellular transmission induces pituitary adenoma resistance to SSTA.** (a) The donor cells (GFP-GH3 or GFP-GH4) treated with OCT (200nM) or PBS (vehicle) for 24h, or their supernatant was added into receptor cells (GH3 or GH4) for co-culture for 24h. (b) Flow cytometry of the expression of GRP78 of co-cultured cells (N=3, ****P<0.0001). (c) The donor cells (GFP-GH3) treated with OCT or PBS (vehicle) for 24h, or their supernatant was added into receptor cells (GH3) with mRFP-GFP labeled LC3 for co-culture for 24h. The autophagosomes were counted under a fluorescence microscope(****P<0.0001). (d) The co-cultured cells were sorted out by flow sorting to separate the receptor cells. (e) Western blot of ERS proteins of separated receptor cells. (f) Western blot of autophagy proteins of separated receptors. (g) Annexin-V apoptosis flow cytometry of separated receptor cells treated with OCT (200nM) for 24h (N=3. GH3: no apoptosis*P=0.0171; early-apoptosis**P=0.0067; late-apoptosis**P=0.0361; GH4: ****P<0.0001). (h) CCK-8 assay of separated receptor (N=5. GH3 ***P=0.0005; GH4 **p=0.0021). (i) ELISA assay of GH concentration of separated receptor cells' supernatant (N=3, *P=0.0172). Data are shown as the mean ± SD of at least three independent experiments. Statistical analyses were conducted using one-way ANOVA and Student's *t* test.

As shown in Fig. 2, our data proved that ERS, acting as resistance signals after SSTA treatment, is unequally distributed in the tumor mass, and according to others' publication 18, we conferred that the ERS signal might transfer from one cell to another to transmit the resistance signals. To prove this, fluorescence-labeled GH3 cells were stimulated with SSTA and intact with non-stimulated tumor cells. The supernatant of the SSTA-treated cells was applied to non-stimulated cells, which were set as the control group (left scheme figure, Fig. 3a). The co-cultured cells were then detected by fluorescence microscope. We observed a significantly higher number of GRP78 positive cell clustering around the “donor cells” (middle and right, Fig. 3a). Moreover, flow cytometry verified the increased population of GRP78 positive pituitary adenoma cells (GH3 and primary tumor cells) after OCT treated “donor cells” directly contacting (Fig. 3b). In addition, we also detected the autophagy after the direct engagement, and observed the remarkable more autophagosomes in GH3 cells exposed to OCT treated “donor cells” (Fig. 3c). To validate further that ERS can be transmitted intercellularly, the “recipient cell” were sorted and examined for ERS associated proteins (Fig. 3d). As expected, “recipient cells” contacted with OCT treated “donor cells” exhibit significantly stronger signals of ERS-associated signatures as well as autophagy markers (Fig. 3e-3f).

To ascertain that the ERS transmission mediates SSTA resistance, the “recipient cells” were further treated with SSTA. Compared to the supernatant group or non-treatment group, “recipient cells” (both GH3 and primary pituitary adenoma cells) contacted with OCT-treated “donor cells” possess notable potent viability, GH release and less apoptosis extent in the presence of SSTA (Fig. 3g-3i, Fig. S4). Moreover, we found that the “recipient cells” that contacted with OCT-treated “donor cells” had higher IC50 of OCT compared to the supernatant group or non-treatment group assessed by CCK-8 assay (Fig. S8). Taken together, these data demonstrated the intercellular transmission of ERS signals promoted SSTA resistance of pituitary adenoma cells.

### IP_3_ mediates intercellular transmission of ERS signal in pituitary adenoma

**Figure 4:**
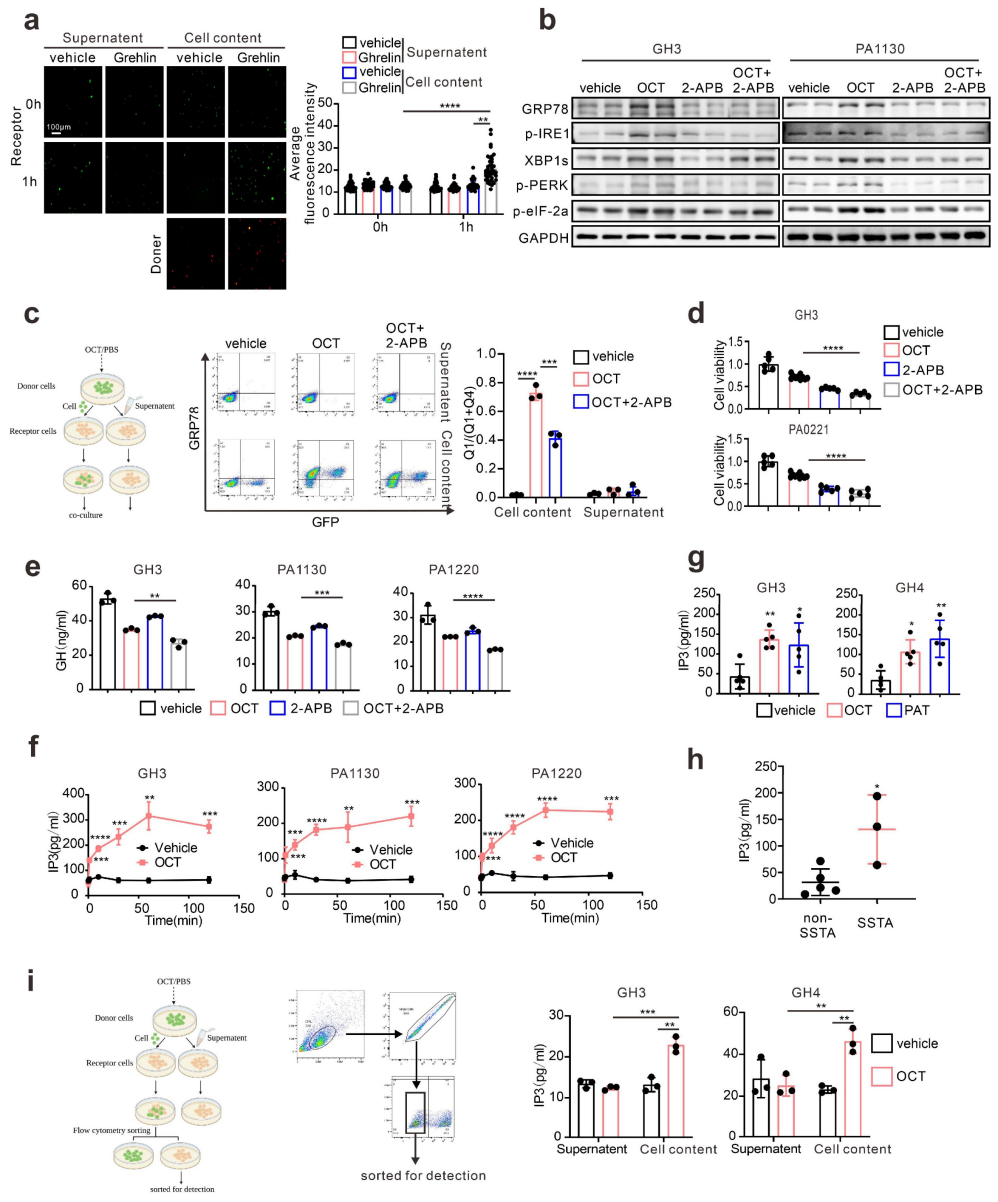
** IP_3_ mediates intercellular transmission of ERS signal in pituitary adenoma.** (a) Calcium imaging of co-cultured cells. The donor cells (GH3) treated with Ghrelin(200μM) or PBS (vehicle) for 24h were incubated with Rhod-2 AM (red fluorescence) for 1h; receptor cells (GH3) were incubated with Fluo-4 AM (green fluorescence) for 1h. Then the donor cells were added into receptor cells for co-culture for 1h. (****P<0.0001; **P=0.005). (b) Western blot of ERS proteins after treatment with either OCT (200nM), 2-APB (100μM), OCT+2-APB, or PBS (vec) for 24h. (c) Flow cytometry of the expression of GRP78 of co-cultured cells. The donor cells (GFP-GH3) treated with OCT (200nM), OCT+2-APB or PBS (vehicle) for 24h, were added into receptor cells (GH3) for co-culture for 24h (N=3. ****P<0.0001; ***P=0.0002). (d) CCK-8 assay of the effect of OCT (200nM), 2-APB (100μM), OCT+2-APB (24h) (N=5, ****P<0.0001). (e) ELISA assay of the effect of OCT (200nM), 2-APB (100μM), OCT+2-APB (24h) on GH concentration of supernatant (N=3. **P=0.005; ***P=0.0009; ****P<0.0001). (f) ELISA assay of the effect of OCT (200nM) on IP3 concentration in cells. (GH3: 1min ***P=0.0004; 10min ****P<0.0001; 30min ***P=0.0008; 60min **P=0.001; 120min ***P=0.0002; PA1130: 1min ***P=0.0004; 10min ***P=0.0005; 30min ****P<0.0001; 60min **P=0.0037; 120min ***P=0.0010; PA1220: 1min ***P=0.0003; 10min ****P<0.0001; 30min ****P<0.0001; 60min ****P<0.0001; 120min ***P=0.0002). (g) ELISA assay of the effect of OCT (30μg/kg.d) and PAT (30μg/kg.d) on IP3 concentration in xenograft cells of nude mice (N=5. GH3: **P=0.0068; *P=0.0188; GH4: *P=0.0182; **P=0.0013). (h) ELISA assay of IP_3_ concentration in GH adenoma primary cell (have used or not used SSTA before operation) (*P=0.0187). (i) ELISA assay of IP_3_ concentration in separated receptor cells. The donor cells (GFP-GH3 or GFP-GH4) treated with OCT or PBS (vehicle) for 24h were added into receptor cells (GH3 or GH4) for co-culture for 24h, the co-cultured cells were sorted out by flow sorting to separate the receptor cells (N=3. GH3: ***P=0.0008; **P=0.0029; GH4: OCT-Cell content vs. OCT-Supernatant**P=0.0086; OCT-Cell content vs. vehicle-Cell content**P=0.0027). Data are shown as the mean ± SD of at least three independent experiments. Statistical analyses were conducted using one-way ANOVA and Student's *t* test.

Next, we intend to understand what molecules mediated ERS intercellular transmission. It has been illustrated that gap junctions, or namely connexins, participate in intercellular signal transduction, including ERS signals 18. Current knowledge concerning connexin indicates that molecules with less than 1.5KD can transmit through connexins, such as calcium and inositol triphosphate (IP_3_) 29. What's more, there are already evidences suggesting that Ca^2+^ signals could be intercellular transmitted via intercellular bridge in both ways of passive Ca^2+^ diffusion and IP_3_-mediated ER Ca^2+^ release 30. Similar to intercellular bridge, gap junctions could also mediate the both transmission forms intercellularly 31. Besides, calcium flow or loss from the endoplasmic reticulum is crucial for ERS, and IP_3_/IP_3_R is considered an essential signal regulating calcium outflow from ER 32. So, IP_3_ may play the role of “messenger” mediating ERS intercellular transmission. Therefore, to testify IP_3_ mediated intercellularly transfer of ERS signal, we first performed calcium imaging on the GH3 cells, which directly contacted with Ghrelin, a classical PLC/IP_3_ activator for pituitary adenoma cells, stimulated tumor cells 33. Indeed, GH3 cells exhibit increased calcium signal when directly contact with Ghrelin stimulated “donor cell” (Fig. 4a). We used OCT as the stimulator, and GH3 cells also exhibit increased calcium signal when directly contact with stimulated “donor cell” (S.5a). To further investigate the role of IP_3_ in ERS signals, IP_3_R inhibitor (2-Aminoethyl diphenylborinate, 2-APB) 34, 35 was used in combination with OCT to treat GH3 cells or primary pituitary adenoma. Our results demonstrated that IP_3_R inhibition remarkably attenuated OCT induced ERS (Fig. 4b). Moreover, results of flow cytometry illustrated that GH3 cells contacted with OCT treated “donor cells” exhibit significant higher population of GRP78 cells, while this effect can be mitigated by IP_3_R blocking (Fig. 4c), and the increase of calcium signal in GH3 cells which directly contact with Ghrelin stimulated “donor cell” as mentioned above was also mitigated by 2-APB (Fig. S5b). Besides, we have also assessed the effect of IP_3_R blockade in combination with SSTA treatment, and synergistic suppression effects were observed on growth or GH release of GH3, GH4 or primary pituitary adenoma cells (Fig. 4d-4e, Fig. S5c-S5d).

Previous studies provide evidence that SSTA could induce intracellular up-regulation of IP_3_ 14, while this was not examined in GH adenoma. Therefore, we assessed the IP_3_ level in pituitary adenoma cells treated with SSTA. As shown in Fig. 4f, IP_3_ level rapidly elevated in GH3 and primary pituitary adenoma cells after OCT stimulation. In addition, a higher level of IP_3_ was also observed in tumor mass of GH3 or GH4 inoculated mice treated with OCT (Fig. 4g). Besides, we have also collected pituitary adenoma surgical resection samples, and IP_3_ level was examined. Consistent with the *in vitro* or* in vivo* data, tumors from patients pre-surgically administrated with OCT expressed significantly higher levels of IP_3_ (Fig. 4h). To examine whether IP_3_ could intercellularly transfer, GH3 cells that direct contact with OCT treated “donor” cells were sorted. We observed notable up-regulation of IP_3_ level of these cells compared to supernatant controls (Fig. 4i). Taken together, we conclude here that SSTA can induce IP_3_ elevation that can intercellularly transmit to neighbor cells, enhancing ERS, which mediate SSTA resistance.

### Connexin 36, instead of Connexin 43, mediates intercellular ERS transmission in pituitary adenoma

**Figure 5:**
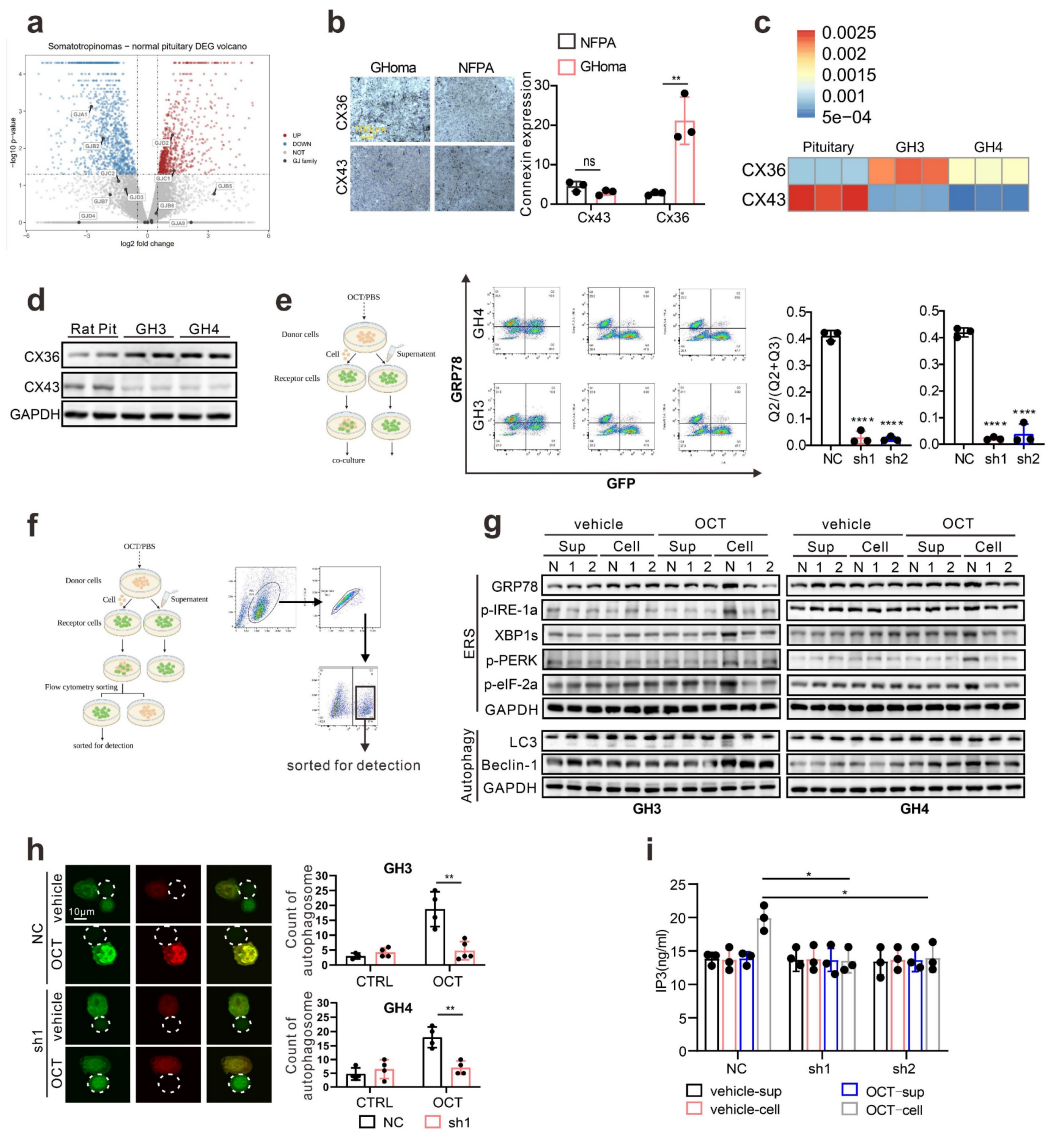
** Connexin 36, instead of Connexin 43, mediates intercellular ERS transmission in pituitary adenoma.** (a) Difference of gene expression between GH adenoma and normal pituitary cells (volcano chart). (b) Immunohistochemical staining of GH adenoma and nonfunctional adenoma tissue sections (N=3, **P=0.0065). (c) The qPCR of expression of CX36 and CX43. The results are shown by heat map (N=3). (d) Western blot of CX43 and CX36 of GH3, GH4 cells and wistar rat pituitary. (e) Flow cytometry analysis of the expression of GRP78 of co-cultured cells. The donor cells (GH3, GH4) were treated with OCT (200nM) for 24h; CX36 of receptor cells were knocked down by shRNA (NC-GH3, sh1-GH3, sh2-GH3; NC-GH4, sh1-GH4, sh2-GH4). The donor cells were added into receptor cells for co-culture for 24h (N=3, ****P<0.0001). (f) The co-cultured cells in(e) were sorted out by flow sorting to separate the receptor cells. (g) Western blot of ERS proteins of separated receptor cells. (h) Laser scanning confocal image of autophagosomes in receptor cells. The donor cells (GFP-GH3) treated with OCT (200nM) or PBS (vehicle) for 24h were added into receptor cells with mRFP-GFP labeled LC3 for co-culture for 24h (GH3: **P=0.0023; GH4: **P=0.0024). (i) ELISA assay of IP_3_ concentration in separated receptor cells (N=3. NC vs sh1*P=0.0137; NC vs sh2*P=0.0221). Data are shown as the mean ± SD of at least three independent experiments. Statistical analyses were conducted using one-way ANOVA and Student's *t* test.

Since connexin 43 was previously reported as an essential channel mediating the ERS transmission 18, we examined the specific connexin in pituitary adenoma. Taking advantage of *in silico* analysis on RNA-seq data (GSE182180), we evaluated the up-regulated genes in GH adenoma from patients compared to normal pituitary tissues (Fig. 5a). Unlike other cell types, connexin 36 (CX 36) up-regulated in GH adenoma, while connexin 43 is significantly down-regulated in GH adenoma (Fig. 5a). To further verify this, we employed immunohistochemistry staining on GH adenomas as wells as non-function pituitary adenomas, and notable stronger signals of CX36 was observed (Fig. 5b). Besides, we have also tested the expression pattern of CX36, CX43 in rat normal pituitary gland and GH3/GH4 cell lines. As shown in Fig. 5c, GH3 and GH4 cells exhibit a much higher level of CX36 compared to normal rat pituitary, unlike CX43. Moreover, we validated the mRNA findings at protein level (Fig. 5d). Together, these results indicate that CX36, instead of CX43, might mediate ERS signal transduction.

To verify that CX36 participating in intercellular ERS signal transduction, we constructed the Cx36 knock-down GH3 and GH4 cell line using CX36 shRNAs (Fig. S6a), and direct co-culture was applied again. GRP78 level in “recipient cells” was determined, and we observed a remarkable reduction of GRP78 expression in both GH3 and GH4 cells (Fig. 5e). To further demonstrate this, the “recipient cells” were sorted by flow cytometry as presented in Fig. 5f, and ERS associated signatures were examined. After OCT pretreated “donor cells” intact co-culture, the up-regulation of ERS-associated proteins in “recipient cells” were significantly attenuated by CX36 knock-down (Fig. 5g). Furthermore, autophagy-related proteins were also examined. Similar findings were revealed (Fig. 5g). To further validate that ERS transmission mediated autophagy was mitigated by CX36, we determined the autophagosome in the neighbored “recipient cells” of “donor cells” and a remarkably decreased number of autophagosome was observed in CX36 knock-down group compared to negative controls (Fig. 5h). Moreover, since our above data implicated that IP_3_ might be the key molecules that responsible for ERS intercellular transmission (Fig. 4), we then also determined the IP_3_ levels in CX36 knock-down “recipient cells”. As expected, CX36 knock-down disrupted the IP_3_ elevation (Fig. 5i). Together, these data suggest that gap junction CX36 mediates intercellular ERS transmission in GH adenoma.

**Figure 6:**
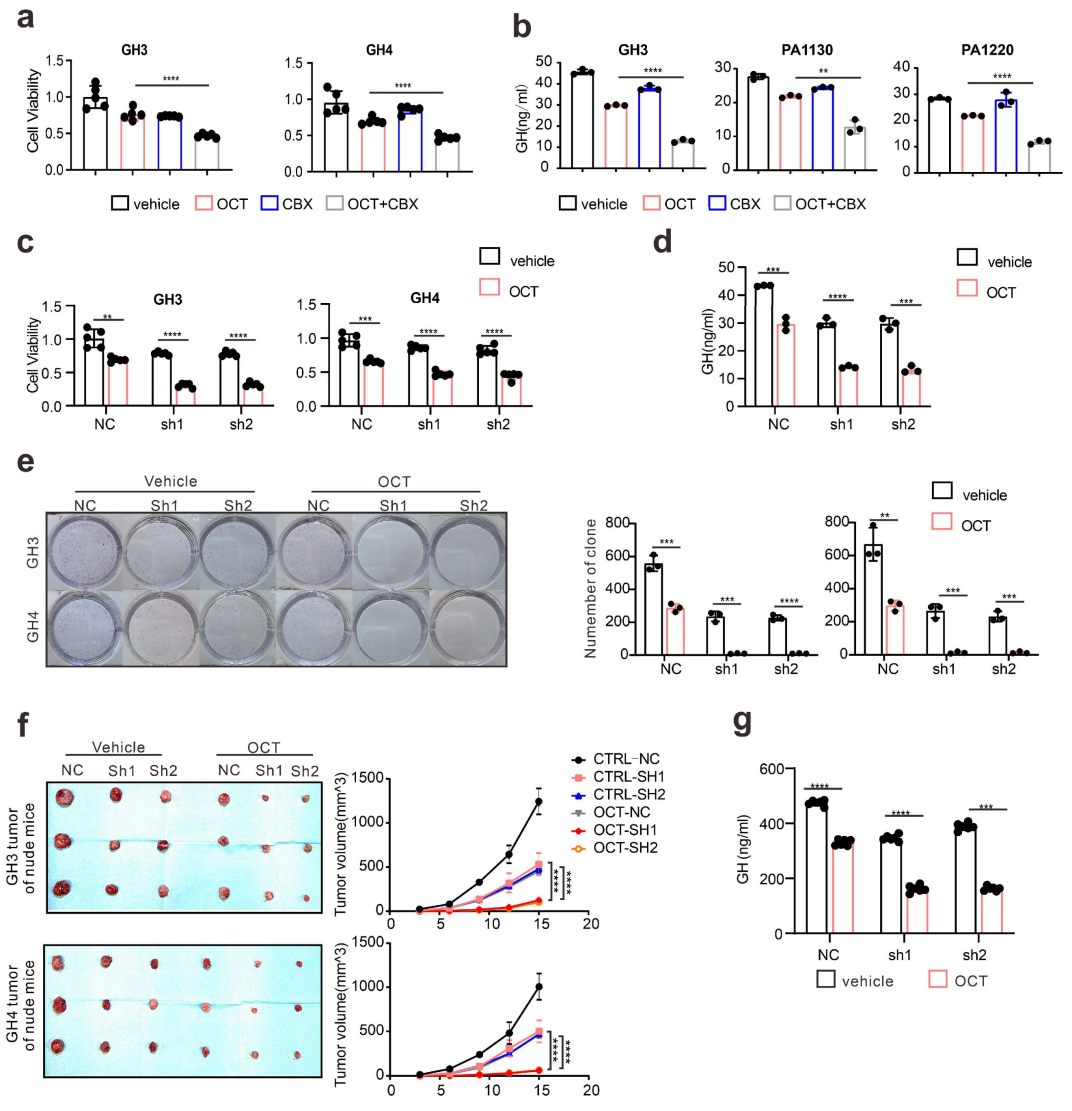
** Connexin 36 mediated intercellular signals transmission de-sensitizes GH adenoma cells to SSTA.** (a) CCK-8 assay of the effect of OCT (200nM), CBX (100μM), OCT+CBX (24h) (N=5, ****P<0.0001). (b) ELISA assay of the effect of OCT (200nM), CBX (100μM), OCT+CBX (24h) on GH concentration in supernatant (N=3. ****P<0.0001; **P=0.0015). (c) CCK-8 assay of the effect of OCT (200nM) (24h) on NC cells or CX36 knock-down cells (N=5. **P=0.0010; ***P=0.0008; ****P<0.0001). (d) ELISA assay of the effect of OCT (200nM) (24h) on GH concentration in supernatant of GH3 cells (NC, sh1, sh2) (N=3. NC: ***P=0.0005; sh1: ****P<0.0001; sh2: ***P=0.0003). (e) Colony formation assay of the effect of OCT (200nM) (24h) on NC cells or CX36 knock-down cells (N=3. GH3: NC **P=0.0036; sh1 ***P=0.0005; sh2 ***P=0.0003; GH4: NC ***P=0.0010; sh1 ***P=0.0002; ****P<0.0001). (f) GH3 and GH4 cells (NC-GH3, sh1-GH3, sh2-GH3; NC-GH4, sh1-GH4, sh2-GH4) were used to form tumors under the axils of nude mice. The nude mice were injected intraperitoneally with either vehicle PBS (vec) or OCT (30μg/kg) once a day. The tumor size was measured every 3 days (volume=0.5*length*width^2) (N=3). (g) ELISA assay of GH concentration in blood samples collected from the orbit of nude mice (N=5, ****P<0.0001). Data are shown as the mean ± SD of at least three independent experiments. Statistical analyses were conducted using one-way ANOVA and Student's *t* test.

Since we hypothesize that ERS intercellular transmission leads to SSTA resistance, to further examine the influence of CX36 on SSTA tumor suppression efficiency, the connexins were pharmaceutically blocked on GH3, GH4 as well as primary pituitary adenoma cells. The viability, colony formation capability, and GH levels were determined. As expected, connexins blocker carbenoxolone (CBX) 36, 37 improved the SSTA tumor suppression effect (Fig. 6a-6b, Fig. S6b-S6c). To specifically examine the role of CX36, the same experiments were also performed on CX36 knock-down GH3 and GH4 cells. Like connexin blockers, CX36 knock-down cells exhibit notably more sensitivity to SSTA-induced tumor inhibition (Fig. 6c-6e). To determine the role of CX36 in pituitary adenoma *in vivo*, we performed the *in vivo* model using CX36 knock-down GH3 and GH4 inoculation and OCT administration. Similar to our *in vitro* data, CX36 knock-down remarkably improved the tumor suppression effect of OCT in GH3 or GH4 models (Fig. 6f-6g, Fig. S6d-S6e). What's more, we found that CX36 expression is much higher in tumor tissues of SSTA-resistant patients than in SSTA-sensitive ones (Fig. S9a). In summary, CX36 disruption can block the ERS signal transduction, affecting SSTA tumor suppression efficiency.

### Quinine, a specific CX36 blocker, sensitize the SSTA tumor suppression effect in pituitary adenoma

**Figure 7:**
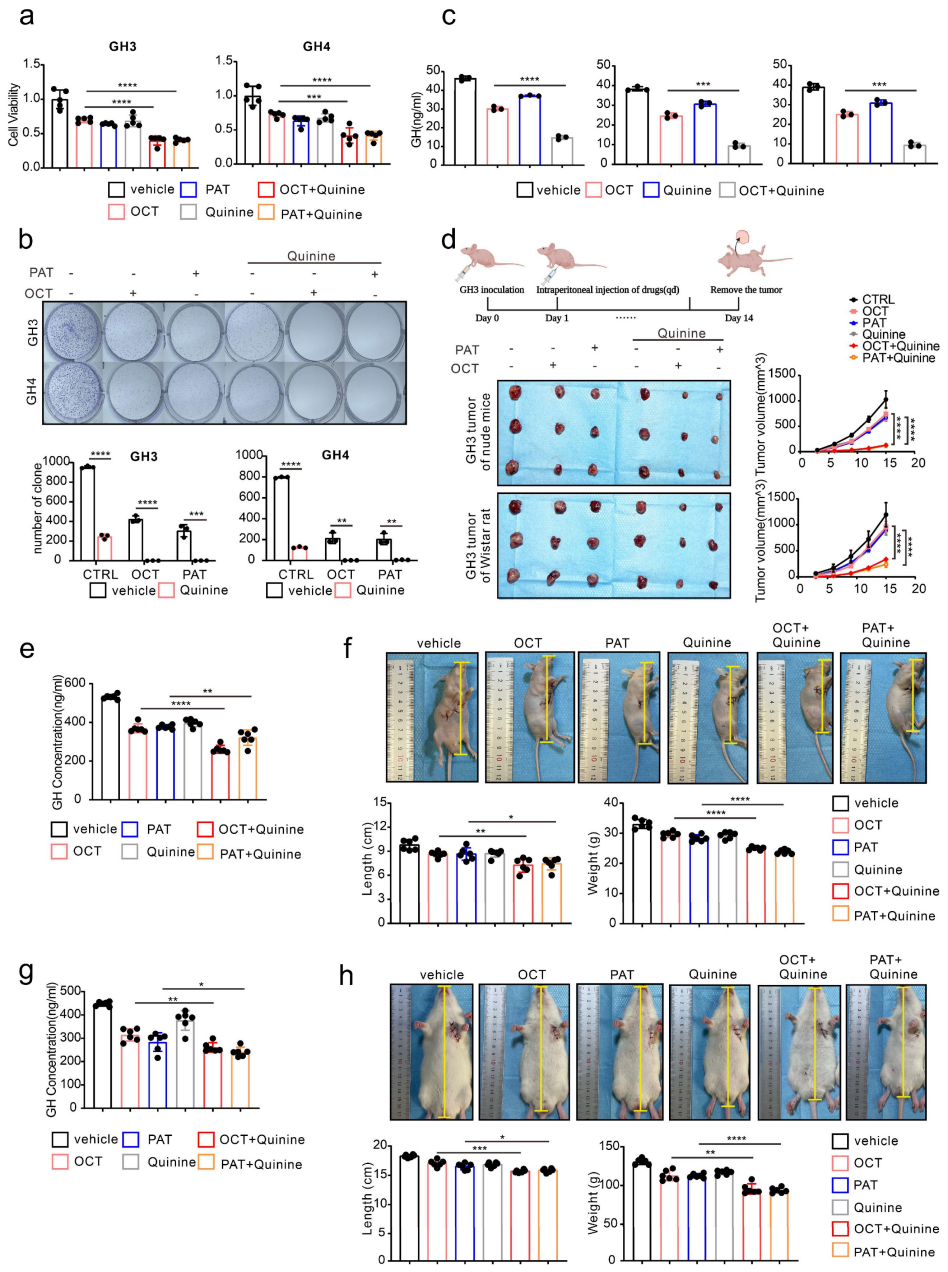
** Quinine, a specific CX36 blocker, sensitizes the SSTA tumor suppression effect in pituitary adenoma.** (a) CCK-8 assay of the effect of OCT (200nM), PAT (200nM), Quinine(100μM), OCT+Quinine, PAT+Quinine (24h) (N=5. ****P<0.0001; ***P=0.0005). (b) Colony formation assay of the effect of OCT (200nM), PAT (200nM), Quinine(100μM), OCT+Quinine, PAT+Quinine (N=3. GH: ****p<0.0001; ***p=0.0010; GH4: ****P<0.0001; OCT: **P=0.0020; PAT: **P=0.0028). (c) ELISA assay of the effect of OCT (200nM), Quinine(100μM), OCT+Quinine (24h) on GH concentration in supernatant (N=5. GH3: ****P<0.0001; PA1130: ***P=0.0001; PA1220: ***P=0.0001). (d) GH3 cells were used to form tumors under the axils of nude mice and wistar rats. The nude mice and wistar rats were injected intraperitoneally with either vehicle PBS (vec), OCT (30μg/kg), PAT (30μg/kg), Quinine (50mg/kg), OCT+Quinine or PAT+Quinine once a day. The tumor size was measured every 3 days (volume=0.5*length*width^2). (N=3, ****P<0.0001). (e) ELISA assay of GH concentration in blood samples collected from the orbit of nude mice (N=6. ****P<0.0001; **P=0.0079). (f) Measure of the body length (**P=0.0046; *P=0.0200), body weight (****P<0.0001) of nude mice (N=6). (g) ELISA assay of GH concentration in blood samples collected from the orbit of wistar rats (N=6. **P=0.0028; *P=0.0425). (h) Measure of the body length (***P=0.0003; *P=0.0268), body weight (**P=0.0010; ****P<0.0001) of wistar rats (N=6). Data are shown as the mean ± SD of at least three independent experiments. Statistical analyses were conducted using one-way ANOVA and Student's *t* test.

Our above data revealed the underlying mechanism of SSTA resistance initiated by ERS intercellular transmission via CX36. Since our ultimate goal is to improve the tumor suppression efficiency of SSTA, we speculated that CX36 blockade might be an ideal therapeutic target. Quinine is an alkaloid derived from the cinchona tree's bark and acts as a safety-specific CX36 blocker, which is also famous for its anti-malaria effect 38, 39. Combined treatment of SSTA and Quinine was applied on GH3 and GH4 cells, and cell viability, colony formation capability, and GH release were determined. Indeed, Quinine and SSTA (both OCT and PAT) exhibit synergistic effects in suppression of tumor growth and hormone release (Fig. 7a-7c). To further verify the synergistic effect* in vivo*, GH3 subcutaneously inoculated nude mice were administrated with SSTA (both OCT and PAT) in combination with Quinine. We observed that Quinine synergized with SSTA to induce the most tumor shrinkage and tumor growth arrest (Fig. 7d, Fig. S7a) indicated by Ki-67 staining (Fig. S7b), and tumor which over-expression CX36 was larger than vector, and the benefit was counteracted by OCT and Quinine (Fig. S9b). Moreover, the suppression of plasma GH level induced by SSTA was also significantly amplified by Quinine administration (Fig. 7e). Furthermore, we evaluated the typical hypertrophy symptoms of acromegaly and revealed that Quinine synergized with SSTA to alleviate hypertrophy in the nude mice acromegaly models (Fig. 7f, Fig. S7c).

To further validate the “sensitize” effect of Quinine in GH adenoma, GH3 cells were subcutaneously injected into wistar rats to better mimic acromegaly. Quinine and SSTA (both OCT and PAT) were co-administrated into the tumor models. As expected, Quinine and SSTA synergistically inhibit the tumor growth and GH level (Fig. S7d-S7e, Fig. 7g). And we evaluated the typical hypertrophy symptoms of acromegaly and revealed that Quinine synergized with SSTA to alleviate hypertrophy in the rat acromegaly models as well (Fig. 7h, Fig. S7f).

It should be noted that first-generation SSTA OCT engages SSTR-2 primarily and SSTR-5 secondarily, second-generation SSTA PAT has an expanded SSTR specificity, especially a higher affinity for SSTR5 40. And approximately 20% of patients with acromegaly who are not sensitive to first-generation SRLs can achieve biochemical control on PAT therapy 9. So, in our study, we need to know whether there is difference between the effects of OCT and PAT on generating endoplasmic reticulum stress and, more importantly, whether there is difference in the mechanisms of resistance to first and second-generation SSTA. We first detected the expression of SSTR2 and SSTR5 in GH3 cells, we observed that both SSTR2 and SSTR5 were expressed on GH3 cells (Fig. S9c). Then, we did western-blot to detect ERS associated proteins of GH3 and GH4 cells after treatment of different concentrations of OCT and PAT. ERS associated proteins were increased by both of OCT and PAT in a concentration-dependent manner (Fig. S9d). Next, the SSTR2 and SSTR5 were knocked down respectively, however, OCT and PAT could still promote the expression of ERS associated proteins (Fig. S9e). And we performed CCK-8 assay, it revealed that there was no significant difference in the inhibitory effects of OCT, PAT and their combination with Quinine on viability of GH3 cells whose SSTR2 or SSTR5 was knocked down (Fig. S9f).

Taken together, our data demonstrated that using Quinine to inhibit CX36 can be a sensitizer to amplify both first and second-generation SSTA-mediated tumor suppression and mitigates symptom of acromegaly.

## Discussion

Somatostatin receptor ligands (SRLs) or Somatostatin analog (SSTA) are the current first-line pharmacological treatment choice for acromegaly 41. Despite the efficient tumor recession achieved, there are still approximately 40% of the patients lacking significant response 8. Therefore, identifying potential mechanisms of SSTA resistance can be beneficial for treating acromegaly patients. Chemotherapy, often leading to danger signals such as oxidative stress or nutrient deprivation, enhance the accumulation of misfolded proteins in the tumor cells 42. Cells reactively emerge a phenomenon called endoplasmic reticulum stress (ERS) that initiates the process named unfolded protein response (UPR) which is identified as removing misfolded proteins 43. Previous studies have demonstrated that ERS suppresses protein synthesis in the early stage and promotes ER homeostasis, which results in rescue of damaged cells 44. Our study, for the first time, illustrated that SSTA elevated the level of ERS and its downstream autophagy in GH adenoma cells, and inhibiting the ERS can further promote SSTA-mediated tumor inhibition. These data uncovered the protective role of ERS in SSTA treatment, which acts as internal resist signals react to SSTA.

There is currently lack of reports illustrating how ERS can be amplified by SSTA treatment. Interestingly, SSTA was revealed as an anti-ERS agent in several inflammations associated studies 45. This difference might be due to the overwhelming effect of inflammation in inducing cellular ERS and SSTA to interfere with pathways related to an inflammatory process. The rapid and long-lasting elevation of IP_3_ in pituitary adenoma cells after SSTA stimulation can lead to the activation of IP_3_R on the ER and further cause calcium loss in the ERS by opening calcium channel. ER calcium loss is an essential signal for initiating ERS attributed to the dysregulation of ER protein folding function 46. Interestingly, early studies found that in some GH adenoma cells, the basic level of IP_3_ was higher in GSP (-) cells 47, and this part of the tumor had a poor response to somatostatin analogs 48, and the early studies of our group showed that the transformation rate of phosphatidylinositols (PI) in some GH adenomas was higher 49, suggesting that IP_3_ plays an essential role in GH adenoma cells.

Next, utilizing the fluorescence labeling SSTA in combination with *in vivo* and 3D *ex vivo* models, we have clarified the uneven distribution of ERS signals within pituitary adenomas, which provides the heterogeneity of ERS in different cells. Therefore, we speculated whether the uneven ERS signals could be transmitted among cells. The biological potentiality that ERS can be transferred intercellularly was recently reported 18. In our present study, taking advantage of intact co-culture of fluorescence cells, flow cytometry sorting, and calcium imaging, we verified that SSTA-induced ERS in pituitary adenoma cells could intercellularly transfer. Hence, this transmission may cause no or less SSTA treated tumor cells to receive stronger level of ERS, leading to less response to SSTA. Connexins were found to be important in transporting signals, including ERS. In the current study, we have characterized that CX36, not CX43, is responsible for ERS transfer. The connexin junctions allow molecules less than 1.5kD, such as IP_3_, Ca^2+^, and cAMP 29, to cross the channel. But there is also a lack of knowledge regarding the mystical molecule that mediates intercellular ERS transmission. Since our data and others proved that SSTA elevated the IP_3_ level in the cells 13, 14, in combination with the conclusion that IP_3_ could transmit intercellularly resulting in Ca^2+^ release from endoplasmic reticulum in the connected cells 30, we then guess that IP_3_ might be the key factor bridging the ERS signal. Indeed, our results proved that SSTA elevated IP_3_ in pituitary adenoma cells, and IP_3_ could be intercellularly transferred. However, our study is unable to provide direct evidence of IP_3_ intercellular transmission among pituitary adenoma cells. Thus, further experiments would be needed to trace IP_3_ flow between cells.

Gap junction (Connexin) family is the transmembrane protein of cellular connection that regulate various cellular functions. Accumulating evidence illustrates connexin's critical role in cellular properties such as invasion, proliferation, metabolic plasticity, and chemoresistance 50-52, but its original ability is to mediate intercellular communication, providing channels for passing small ions and metabolites less than 1.5kD 29. Connexin 43 (CX43) is the most widely investigated member of connexins. The loss of connexins, such as CX43, can suppress or facilitate tumor growth depending on diverse cancer types 53, 54. Our study proved the functional CX36 instead of CX43 in mediating ERS transmission in GH adenoma cells. These observations raised our interest in validating the potential therapeutic effect of Quinine, a CX36-specific blockade and classic anti-malaria drug 38, 39. Our data provide evidence of the efficient sensitization effect of Quinine in SSTA treatment, which can potentially translate into clinical usage due to its safety and efficiency.

In conclusion, our present study uncovers the intrinsic SSTA resistance signals mediated by ERS intercellular transmission in pituitary adenoma and can be blocked by CX36 inhibition. These results may provide novel insight into chemoresistance and therapeutic targets for improving SSTA efficiency in GH adenoma treatment.

## Supplementary Material

Supplementary methods and figures.

## Abbreviations

2-APB: 2-aminoethyl diphenylborinate
4-PBA: 4-Phenylbutyric acid
CBX: carbenoxolone
CNS: central nerve system
CX: connexin
ERS: endoplasmic reticulum stress
GH: growth hormone
GHoma: growth hormone adenoma
GPCR: G protein-coupled receptor
IP_3_: inositol triphosphate
NFPA: non-functional pituitary adenoma
OCT: octreotide
PAT: pasireotide
PI: phosphatidylinositol
PLC: phospholipase C
SSTA: somatostatin analog
SRLs: somatostatin receptor ligands
SSTR: somatostatin receptor
Tm: tunicamycin
