# Supplementary material for: Connexin 36 Mediated Intercellular Endoplasmic Reticulum Stress Transmission Induces SSTA Resistance in Growth Hormone Pituitary Adenoma
Source: Int J Biol Sci. 2024 Jan 1;20(2):801–17. doi: 10.7150/ijbs.86736 (PMC10758099; doi:10.7150/ijbs.86736)
Supplement: Supplementary file 1 — Supplementary methods and figures. [file ijbsv20p0801s1.pdf]

Rat pituitary extracts

The anesthetized rat was fixed on the plate; the neck hair was cut off, and the local area was routinely disinfected. The animal was made a median cervical incision and blunt separation of the subcutaneous fascia and submandibular gland until the sternohyoid muscle was exposed. The muscular layer was separated downward and inward from the lateral edge of the sternohyoid muscle to the median line of the skull base to find the occipital crest. The muscles attached to the occipital bone were stripped from the occipital crest and extended upward to separate the suture of the occipital sphenoid, and the cranial area was drilled at the intersection of the occipital crest and the occipital sphenoid suture. Adjust the core of the skull drilling needle, which is about longer than 0.5mm of the needle tooth, and fix the needle core at the intersection of the occipital crest and the occipital sphenoid suture, drill down vertically, and immediately postscript the drilling needle when the sensory bone plate is drilled through, and the resistance decreases. Remove the bleeding from the drill with a small cotton ball, pick out the outer and internal bone plates with a long straight needle with a hook or clip the outer and internal bone plates with straight-pointed ophthalmic tweezers, use a triangular needle to break the meninges of the pituitary gland, and then use a glass tube connected to the 1/8 horsepower attractor to suck out the pituitary gland. After the operation, close and the disinfect wound. And then, the pituitaries were dissected into two halves for protein and RNA extraction, respectively.

Western blot

Protein concentrations of cells and animal tissue were determined by using a BCA kit. Then the protein samples were diluted with 5x sample buffer solution, separated by electrophoresis in a 12% separation gel for 90 min, and blocked with 1× PBS containing 5% non-fat dried milk for 1 h at room temperature. Then, the cells were incubated with primary Antibodies: GRP-78(1:1000, Proteintech, Wuhan, China); P-IRE-1α(1:1000, Abclonal, Wuhan, China); P-PERK(1:1000, Abclonal, Wuhan, China); XBP-1S(1:1000, Proteintech, Wuhan, China); P-eIF-2α(1:1000, Abclonal, Wuhan, China); Beclin-1(1:1000, Proteintech, Wuhan, China); LC-3(1:1000, Cell Signaling Technology, Beverly, MA, USA); CX43(1:1000, Abclonal, Wuhan, China); CX36(1:1000, Abclonal, Wuhan, China) and GAPDH(1:5000, Proteintech, Wuhan, China)at 4 °C overnight. The membranes were washed and incubated with HRP-conjugated anti-rabbit or mouse antibody (1:5000, Proteintech, Wuhan, China) for 2h at room temperature and then exposed and photographed on a Gene Gnome exposure instrument. Finally, the expression of the proteins was standardized for densitometric analysis to GAPDH levels.

Immunofluorescence staining

Cells or tissue sections were incubated with GRP-78(1:200, Proteintech, Wuhan, China); P-IRE-1α(1:200, Abclonal, Wuhan, China); P-PERK(1:200, Abclonal, Wuhan, China); XBP-1S(1:200, Proteintech, Wuhan, China); P-eIF-2α(1:200, Abclonal, Wuhan, China) and CD31(1:200, abcam, Cambridge, United Kingdom) antibodies overnight at 4°C and then incubated with conjugated secondary antibody for 2h at room temperature

in the dark. After several washes with PBST, the slides were incubated with DAPI for 30 mins and then mounted in glycerol. After mounting, immunofluorescent signaling was observed with an Olympus Fluoview laser scanning confocal microscope (Olympus, Tokyo, Japan). The percentages of positive cells were counted in a blinded manner using ImageJ.

Quantitative real-time PCR

Total RNA was isolated from the cells or tissue using TRIzol (Servicebio, Wuhan, China) before being washed with PBS and reverse-transcribed to cDNA with the Hifair® III 1st Strand cDNA Synthesis Kit (Yeasen, Shanghai, China) according to the datasheet from the manufacturer. Gene products were then amplified by quantitative real-time PCR on an ABI-Prism 7500 Real-Time PCR System (Applied Biosystems, Carlsbad, CA, USA) using Hieff® qPCR SYBR Green Master Mix (Low Rox Plus) (Yeasen, Shanghai, China). The mRNAs were normalized to the internal standard GAPDH. Data were analysed using the $2^{(-\Delta\Delta Ct)}$ method. The primers are as follows:

CX43(Forward: CTGGAGAAACCTGCCAAGTATG, Reverse: GGTGGAAGAATGGGAGTTGCT);

CX36(Forward: TGGGAGCAAGCGAGAAGATAA, Reverse: GGATGATGTAGAAGCGGGAAAT);

GAPDH (Forward: CTGGAGAAACCTGCCAAGTATG, Reverse: GGTGGAAGAATGGGAGTTGCT).

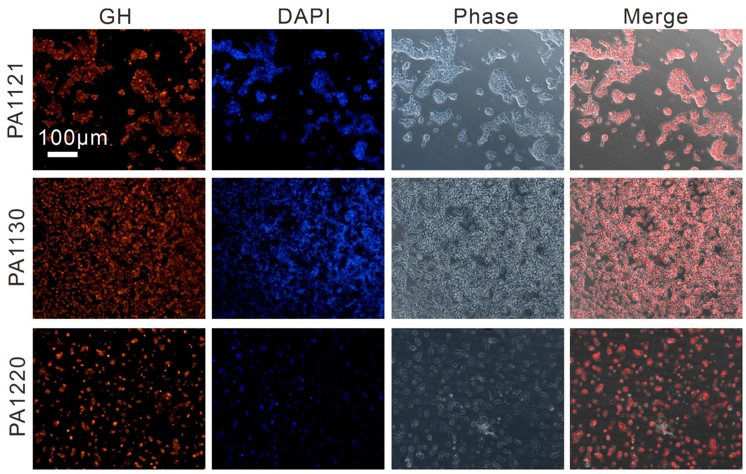

Figure S1. Immonofluorescence staining of human GH adenoma cells.

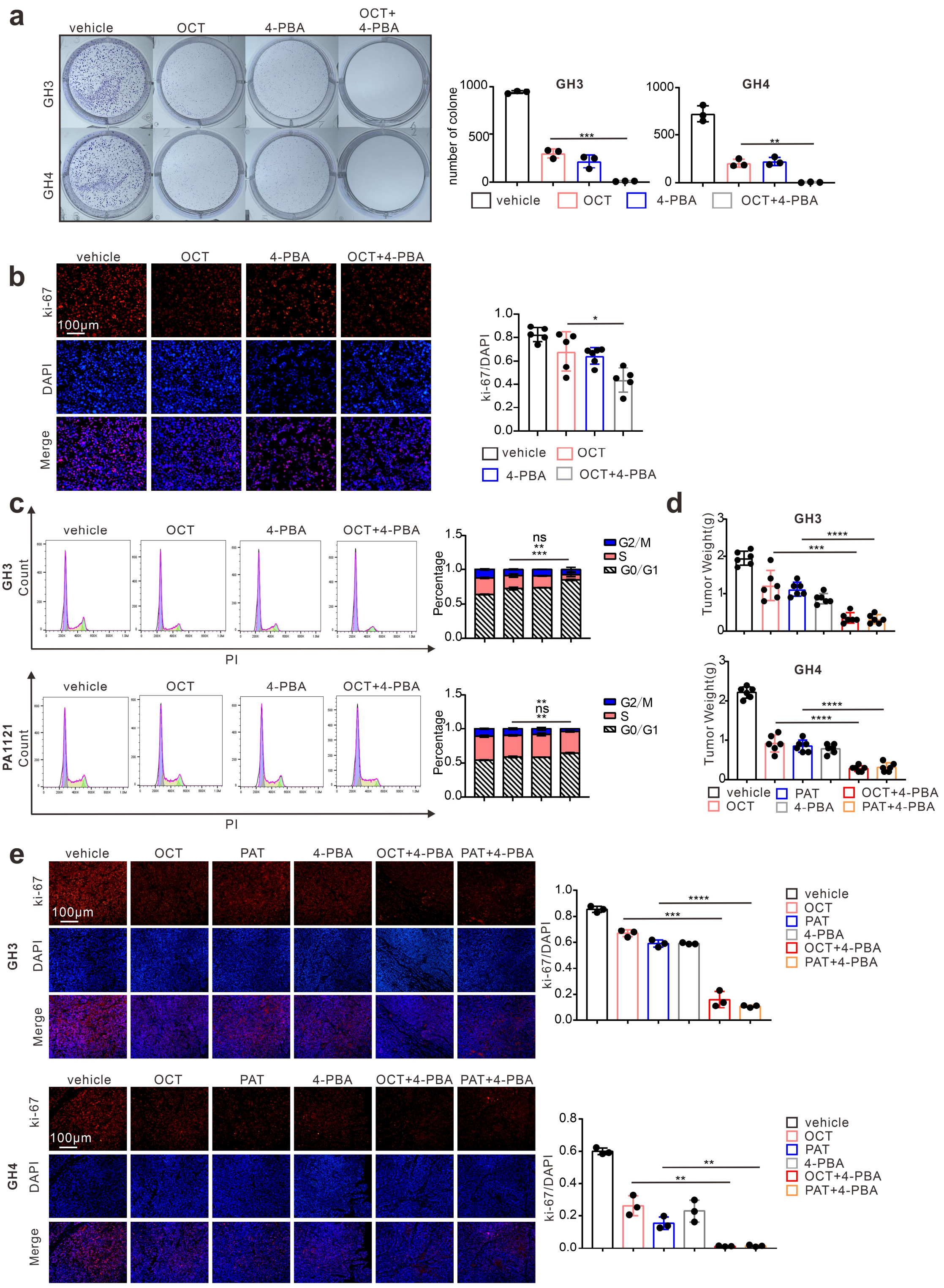

Figure S2. (a) Colony formation assay of the effect of OCT (200nM), 4-PBA (500μM), OCT+4-PBA (24h) (N=3. ***P=0.0001; **P=0.0025).

(b) Ki-67 assay of GH3 cells treated with either octreotide (OCT) (200nM),4-PBA (500μM), OCT+4-PBA or PBS (vehicle) for 24h (N=5, *p=0.0248).

(c) PI cell cycle flow cytometry of GH3 and human GH adenoma primary cells treated with OCT (200nM), 4-PBA (500μM), OCT+4-PBA or PBS (vehicle) for 24h (N=3. GH3: ***p=0.0006; **p=0.0065; PA1121:G0/G1**P=0.0025; G2/M**P=0.0040).

(d) Tumor weight of nude mice (N=3. ***P=0.0005; ****P<0.0001).

(e) Ki-67 Immonofluorescence staining of sections of nude mice tumor (N=3. GH3: ***P=0.0002; ****P<0.0001; GH4: OCT vs OCT+4-PBA**P=0.0023; PAT vs PAT+4-PBA**P=0.0033)

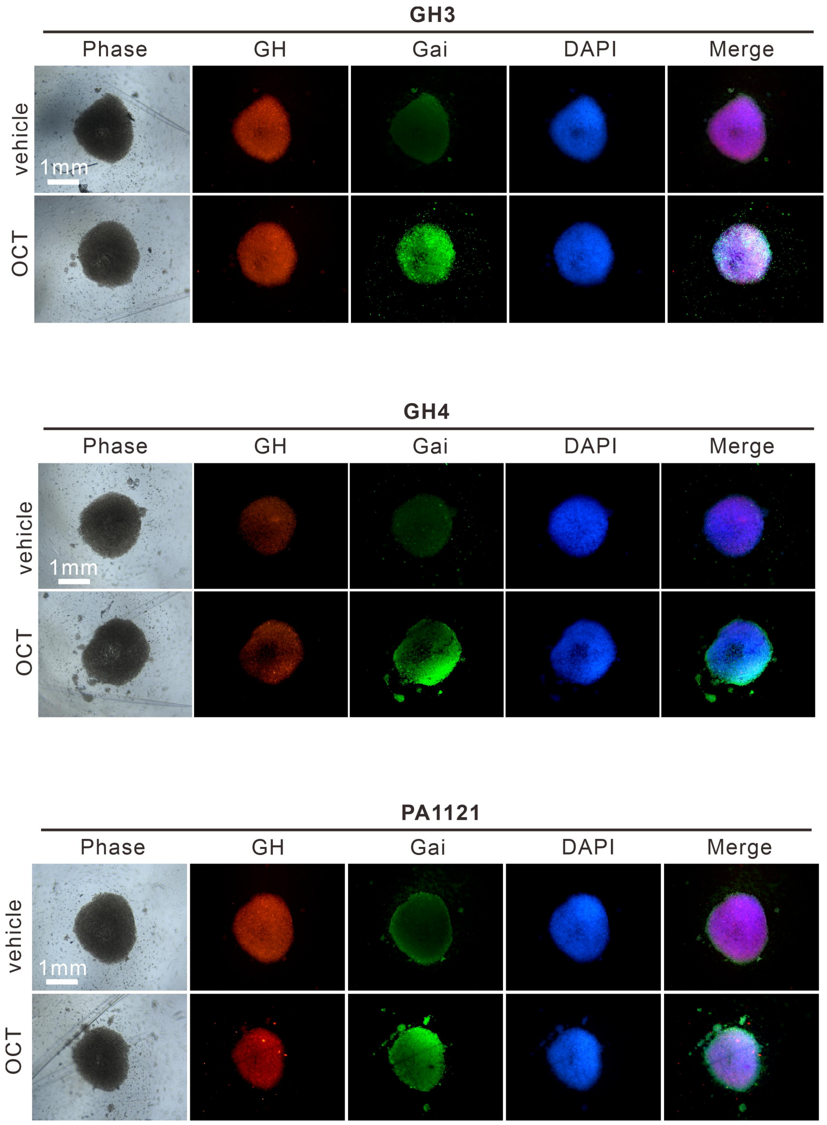

Figure S3. Immunofluorescence staining of 3D spheres.

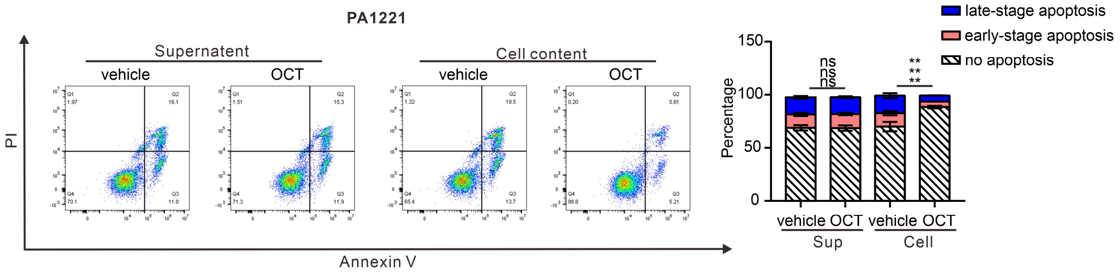

Figure S4. Annexin-V apoptosis flow cytometry of separated receptor cells (human primary pituitary adenoma cells) treated with OCT (200nM) for 24h (N=3. no apoptosis**P=0.0020; early-apoptosis**P=0.0031; late-apoptosis**P=0.0011).

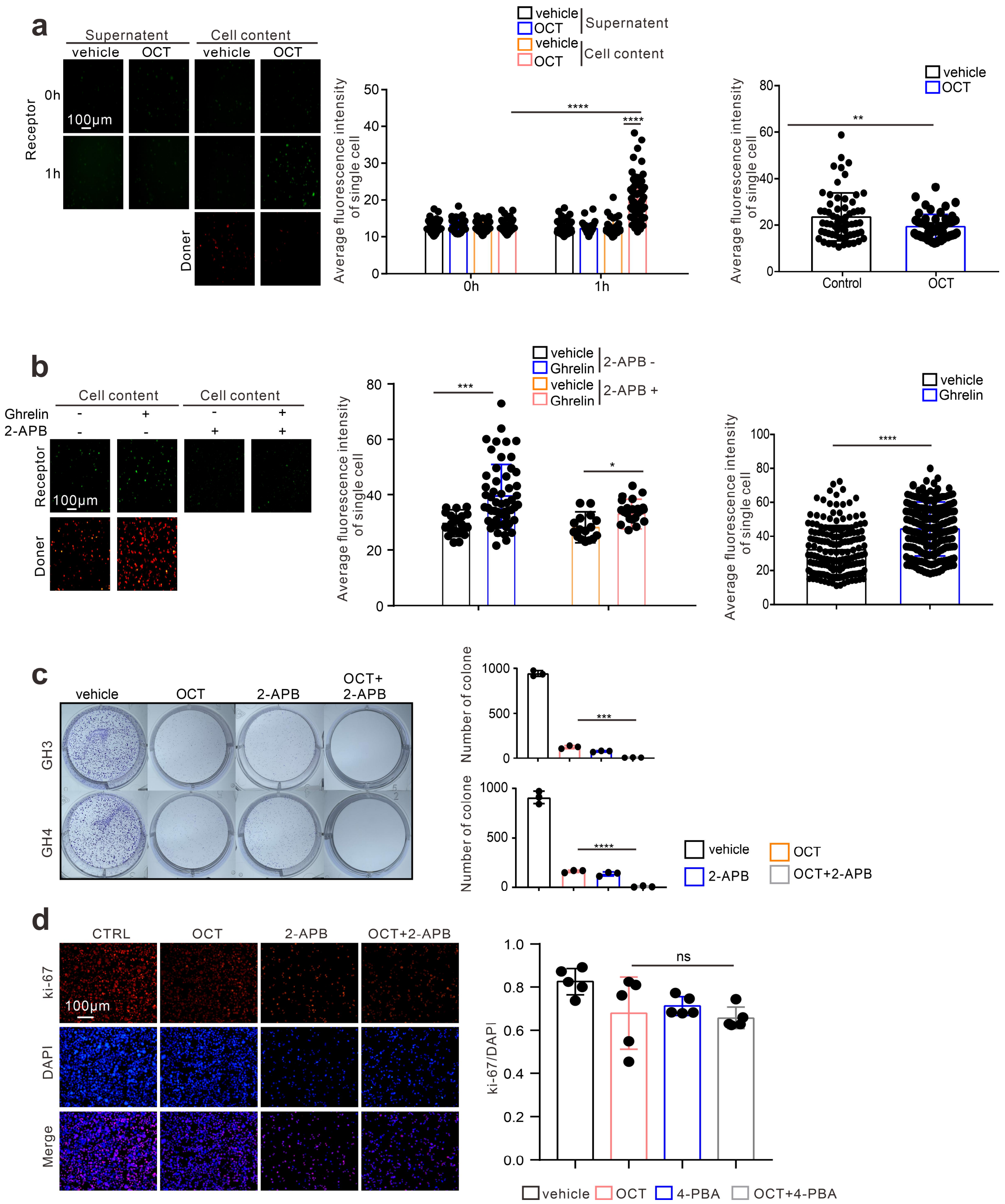

Figure S5. (a) Calcium imaging of co-cultured cells. The donor cells (GH3) treated with OCT (200nM) or PBS (vehicle) for 24h were incubated with Rhod-2AM (red fluorescence) for 1h; receptor cells (GH3) were incubated with Fluo-4AM (green fluorescence) for 1h. Then the donor cells were added into receptor cells for co-culture for 1h (****P<0.0001; **P=0.0089).

(b) Calcium imaging of co-cultured cells. The donor cells (GH3) treated with Ghrelin (200μM) or PBS (vehicle) for 24h were incubated with Rhod-2AM (red fluorescence) for 1h, receptor cells (GH3) were treated with 2-APB (100μM) or PBS (vehicle) for 24h, and were incubated with Fluo-4AM (green fluorescence) for 1h. Then the donor cells were added into receptor cells for coculture for 1h (***P=0.0001; *P=0.0103; ****P<0.0001).

(c) Colony formation assay of the effect of OCT (200nM), 2-APB (100μM), OCT+2-

APB (N=3. ***P=0.0003; ****P<0.0001).

(d) Ki-67 assay of GH3 cells treated with either octreotide (OCT) (200nM),2-APB (100μM), OCT+2-APB or PBS (vehicle) for 24h (N=3).

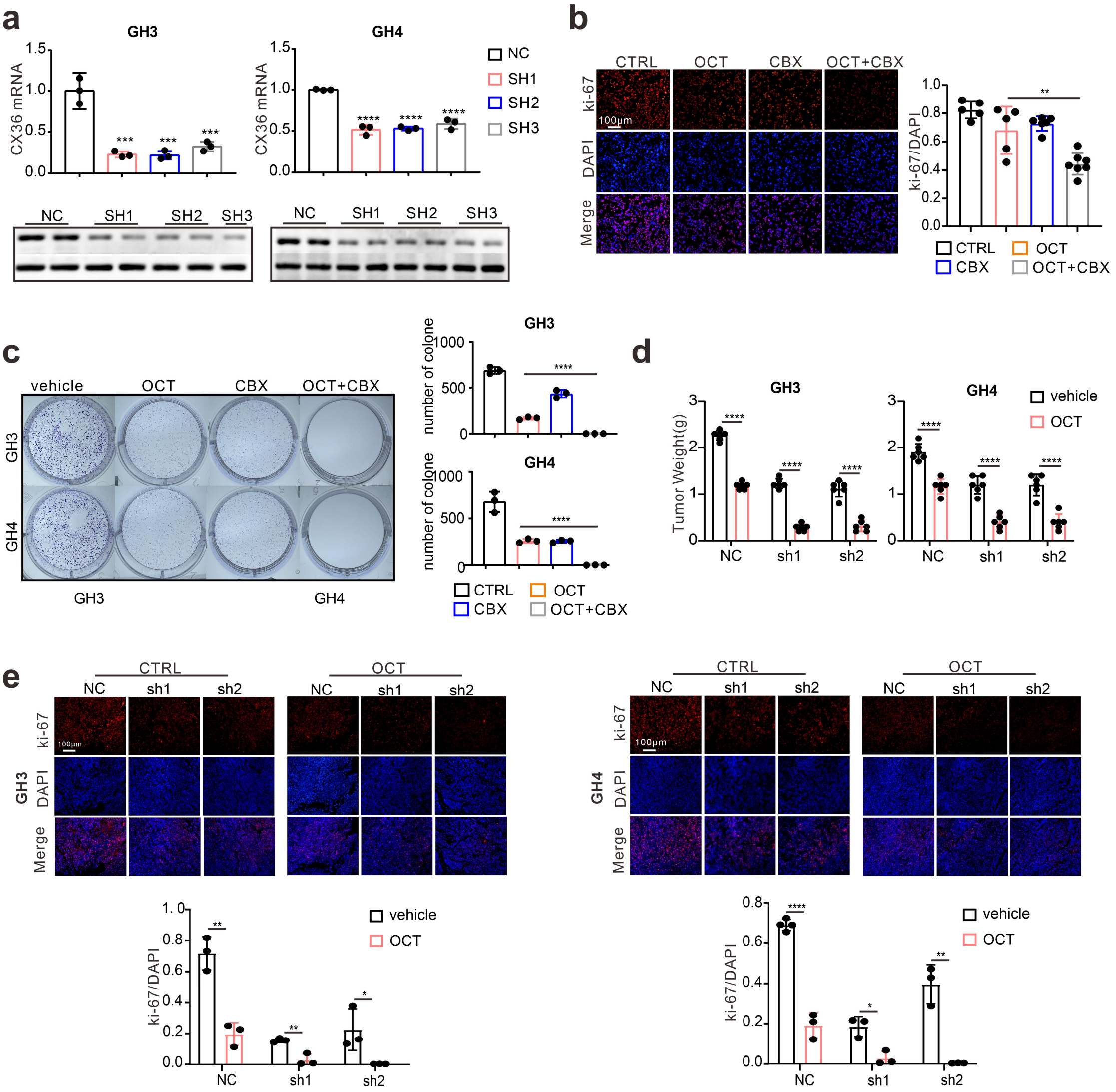

Figure S6. (a) Verification of knockdown of CX36 (qPCR and western-blot).

(b) Ki-67 assay of GH3 cells treated with either octreotide (OCT) (200nM), CBX (100μM), OCT+CBX or PBS (vehicle) for 24h (N=3, **p=0.0075).

(c) Colony formation assay of the effect of OCT (200nM), CBX (100μM), OCT+CBX (N=3, ****P<0.0001).

(d) Tumor weight of nude mice (N=6, ****P<0.0001).

(e) Ki-67 Immonofluorescence staining of sections of nude mice tumor (GH3: NC**P=0.0023; sh1**P=0.0060; sh2*P=0.0453; GH4: NC****P<0.0001; sh1*P=0.0109; sh2**P=0.0021).

Data are shown as the mean ± SD of at least three independent experiments. Statistical analyses were conducted using one-way ANOVA and Student's t test.

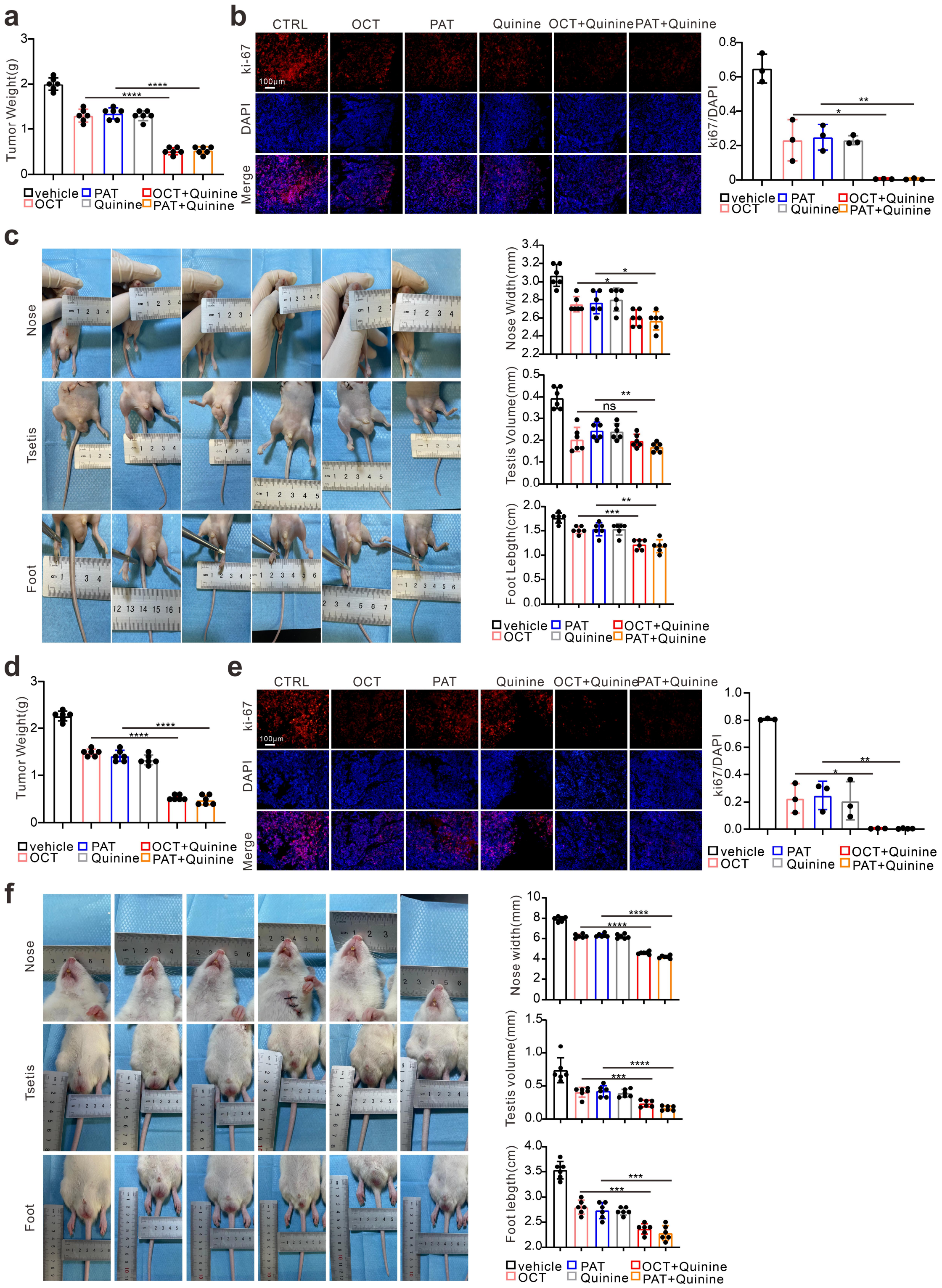

Figure S7. (a) Tumor weight of nude mice (N=6, ****P<0.0001).

(b) Ki-67 immonofluorescence staining of sections of nude mice tumor (*P=0.0306; **P=0.0049).

(c) Nose width of nude mice (N=6. OCT vs OCT+Quinine*P=0.0133; PAT vs PAT+Quinine*P=0.0117); Testis volume of nude mice (N=6, **P=0.0018); Foot length of nude mice (N=6. ***P=0.0001; **P=0.0011).

(d) Tumor weight of Wistar rats (N=6, ****P<0.0001).

(e) Ki-67 Immonofluorescence staining of sections of wistar rat tumors (*P=0.0238; **P=0.0047).

(f) Nose width of Wistar rats (N=6, ****P<0.0001); Testis volume of wistar rats (N=6. ***P=0.0010; ****P<0.0001); Foot length of Wistar rats (N=6. OCT vs OCT+Quinine***P=0.0001; PAT vs PAT+Quinine***P=0.0005).

Data are shown as the mean ± SD of at least three independent experiments. Statistical analyses were conducted using one-way ANOVA and Student's *t* test.

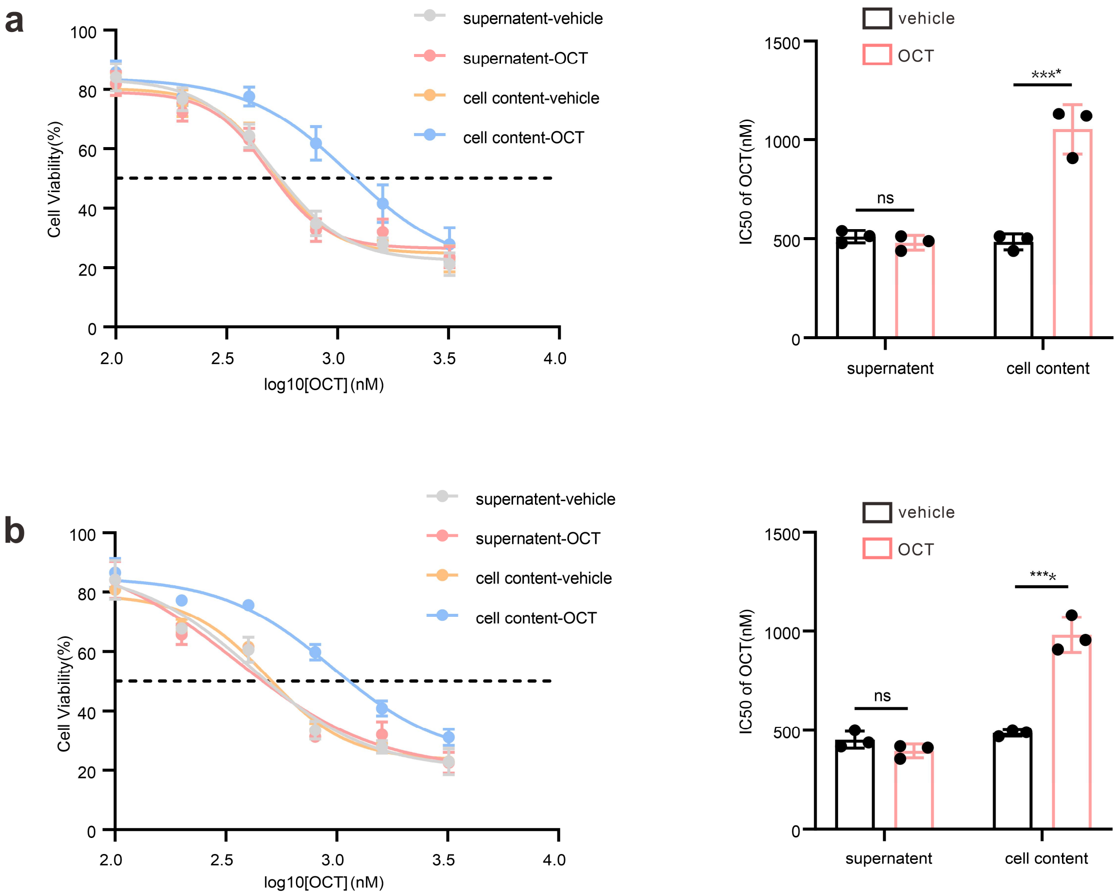

Figure S8. Dose-response curves of GH3 cells (a) and primary growth hormone adenoma cells (b) to OCT by CCK-8 assay and compared the IC50 of each group of cells (****P<0.0001).

Data are shown as the mean ± SD of at least three independent experiments. Statistical analyses were conducted using Student's *t* test.

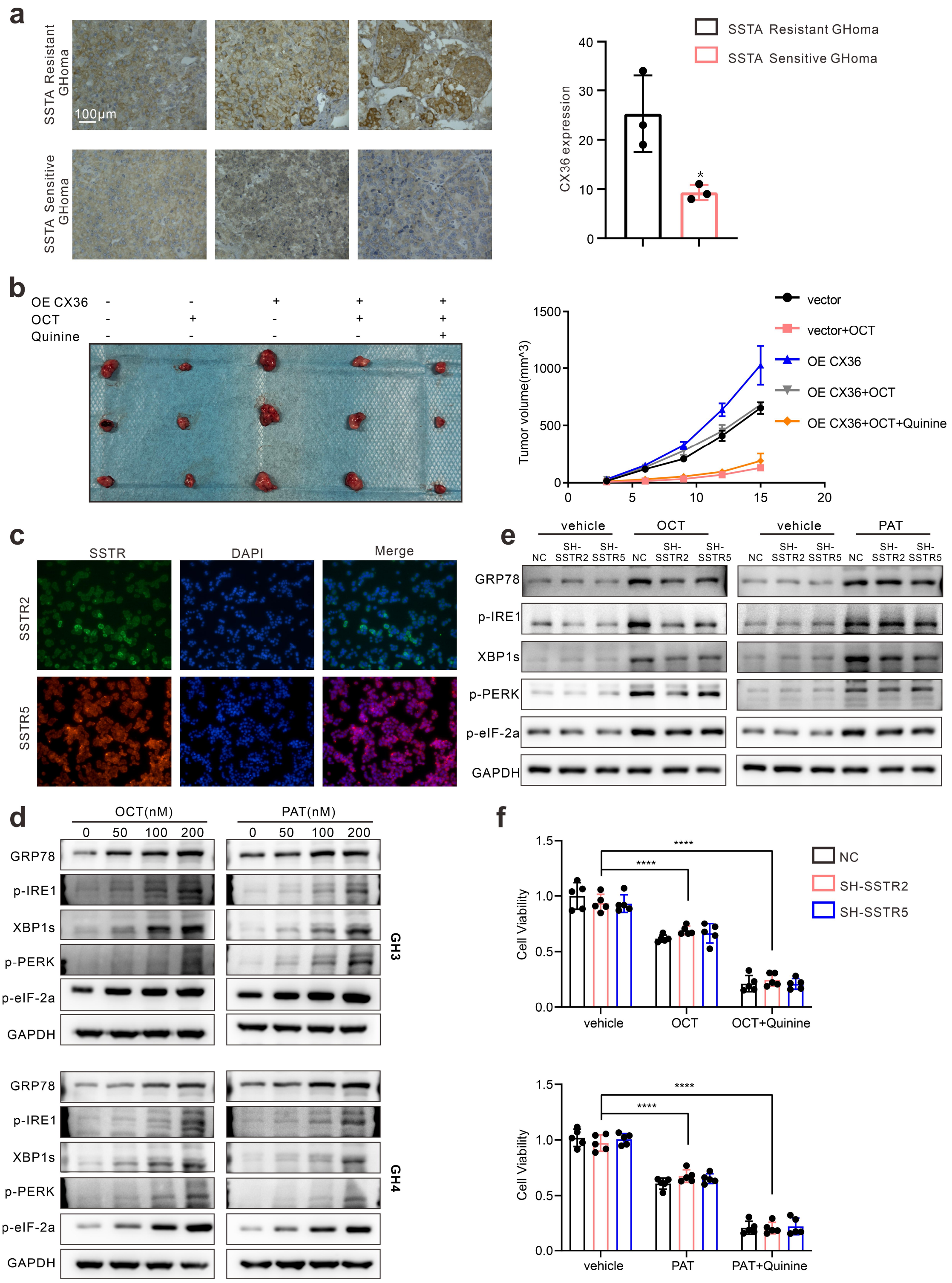

Figure S9. (a) Immunohistochemical staining of CX36 in SSTA-resistant and SSTA-sensitive GH adenoma tissue sections (N=3, *P=0.0249).

(b) GH3 cells were used to form tumors under the axils of nude mice, OCT (30μg/kg) and Quinine (50mg/kg) were injected intraperitoneally for once a day. The tumor sizes were measured every 3 days (volume=0.5*length*width^2) (N=3).

(c) Immonofluorescence staining of GH3 cells.

(d) Western blot of ERS proteins of GH3 and GH4 cells after treatment with different concentration of OCT or PAT for 24h.

(e) Western blot of ERS proteins of GH3 cells (NC, SH-SSTR2 and SH-SSTR5) after treatment with either vehicle (PBS) or OCT (200nM) for 24h.
(f) CCK-8 assay of the effect of OCT (200nM) and OCT+Quinine (100μM) on GH3 cells (NC, SH-SSTR2 and SH-SSTR5) (N=5, ****$P<0.0001$).
Data are shown as the mean ± SD of at least three independent experiments. Statistical analyses were conducted using one-way ANOVA and Student's *t* test.
